# Unraveling an Alternative Mechanism in Polymer Self-Assemblies: An Order–Order Transition with Unusual Molecular Interactions between Hydrophilic and Hydrophobic Polymer Blocks

**DOI:** 10.1021/acsnano.3c00722

**Published:** 2023-03-27

**Authors:** Lukas Hahn, Theresa Zorn, Josef Kehrein, Tobias Kielholz, Anna-Lena Ziegler, Stefan Forster, Benedikt Sochor, Ekaterina S. Lisitsyna, Nikita A. Durandin, Timo Laaksonen, Vladimir Aseyev, Christoph Sotriffer, Kay Saalwächter, Maike Windbergs, Ann-Christin Pöppler, Robert Luxenhofer

**Affiliations:** † Institute for Functional Materials and Biofabrication, Department of Chemistry and Pharmacy, Julius-Maximilians-University Würzburg, Röntgenring 11, 97070 Würzburg, Germany; ‡ Institute of Pharmacy and Food Chemistry, Department of Chemistry and Pharmacy, Julius-Maximilians-University Würzburg, Am Hubland, 97074 Würzburg, Germany; § Center for Nanosystems Chemistry & Institute of Organic Chemistry, Department of Chemistry and Pharmacy, Julius-Maximilians-University Würzburg, Am Hubland, 97074 Würzburg, Germany; ∥ Institute of Pharmacy and Food Chemistry, Department of Chemistry and Pharmacy, Julius-Maximilians-University Würzburg, Am Hubland, 97074 Würzburg, Germany; ⊥ Institute of Pharmaceutical Technology and Buchmann Institute for Molecular Life Sciences, Goethe University Frankfurt, Max-von-Laue-Str. 9, 60438 Frankfurt am Main, Germany; # Chair for X-Ray Microscopy, Julius-Maximilians-University Würzburg, Josef-Martin-Weg 63, 97074 Würzburg, Germany; ∇ Faculty of Engineering and Natural Science, Tampere University, Korkeakoulunkatu 8, 33720 Tampere, Finland; ○ Division of Pharmaceutical Biosciences, Faculty of Pharmacy, University of Helsinki, Viikinkaari 5 E, 00014 Helsinki, Finland; ◆ Soft Matter Chemistry, Department of Chemistry, Helsinki Institute of Sustainability Science, Faculty of Science, University of Helsinki, 00014 Helsinki, Finland; ¶ Institute of Physics-NMR, Martin-Luther-Universität Halle-Wittenberg, Betty-Heimann-Str. 7, 06120 Halle, Germany

**Keywords:** inverse thermogelation, poly(2-oxazoline), poly(2-oxazine), molecular dynamics simulation, NMR spectroscopy

## Abstract

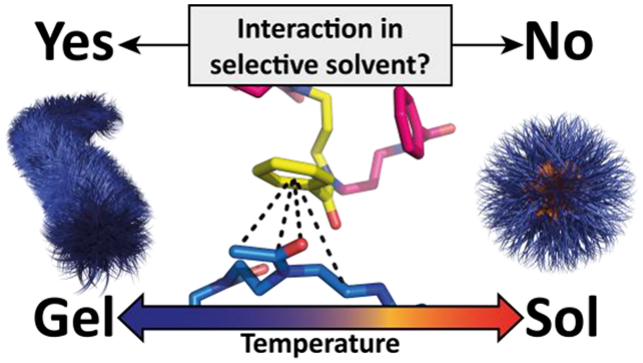

Polymer self-assembly leading to cooling-induced hydrogel formation is relatively rare for synthetic polymers and typically relies on H-bonding between repeat units. Here, we describe a non-H-bonding mechanism for a cooling-induced reversible order–order (sphere-to-worm) transition and related thermogelation of solutions of polymer self-assemblies. A multitude of complementary analytical tools allowed us to reveal that a significant fraction of the hydrophobic and hydrophilic repeat units of the underlying block copolymer is in close proximity in the gel state. This unusual interaction between hydrophilic and hydrophobic blocks reduces the mobility of the hydrophilic block significantly by condensing the hydrophilic block onto the hydrophobic micelle core, thereby affecting the micelle packing parameter. This triggers the order–order transition from well-defined spherical micelles to long worm-like micelles, which ultimately results in the inverse thermogelation. Molecular dynamics modeling indicates that this unexpected condensation of the hydrophilic corona onto the hydrophobic core is due to particular interactions between amide groups in the hydrophilic repeat units and phenyl rings in the hydrophobic ones. Consequently, changes in the structure of the hydrophilic blocks affecting the strength of the interaction could be used to control macromolecular self-assembly, thus allowing for the tuning of gel characteristics such as strength, persistence, and gelation kinetics. We believe that this mechanism might be a relevant interaction pattern for other polymeric materials as well as their interaction in and with biological environments. For example, controlling the gel characteristics could be considered important for applications in drug delivery or biofabrication.

The characteristics that arise from hydrogelation of aqueous polymeric solutions include their high water content, porous structure, and tunable physicochemical properties.^1,2^ Precisely, these make hydrogels particularly suitable for various biomedical applications,^3,4^ where the ability to (reversibly) trigger gelation in response to, for example, temperature around physiological temperatures is possible. Such environmentally responsive features are a key property for smart biomaterials.^5,6^

Thermogelation upon heating^7,8^ is well-known in the literature^9,10^ and structurally diverse thermogelling block copolymers are plentiful with Pluronic F127 being arguably the most prominent example.^11^ Mostly, thermogelation relies on a thermally triggered disorder–order transition from random coils to polymer micelles forming dense colloidal packings.^7,12^ In contrast, only a few examples of thermogelation through order–order transitions are currently known. In 2012, Armes and co-workers described a thermogelling system based on a heating induced sphere-to-worm order–order transition,^13^ which could be tuned with respect to the critical gelation temperature.^14^ Later, Penfold *et al*. introduced a system that combined a pH-responsive vesicle-to-worm transition and a thermoresponsive sphere-to-worm transition.^15^ More recently, a thermogelling diblock copolymer was presented, which formed spheres (4 °C, weakly turbid free-flowing fluid), worms (22 °C, turbid free-standing gel), or vesicles (50 °C, milky-white free-flowing dispersion) in aqueous solution.^16^

While inverse thermogelation (gelation upon cooling) of polymers is well-known for bio- or bioderived polymers such as agarose or gelatin, only a few of such systems based on synthetic polymers are described in the literature.^17^ Arguably the best known synthetic inverse thermogelling polymer is poly(*N*-acryloyl glycinamide) (PNAGA), which was introduced by Haas *et al*. as early as 1967.^18^ Later on, further examples for inverse thermogelling systems were reported by Fu *et al*.^19,20^ and Parmar *et al*.^21^ What these systems have in common is that inter- and intramolecular hydrogen bonds or electrostatic interactions between polymer repeat units are formed upon cooling, which is a main driving force for the gelation.

Very recently, we described an ABA-type block copolymer, which shows an unusual, reversible, cooling-induced sphere-to-worm order–order transition along with (inverse) thermogelation (Figure 1).^22^ The corresponding amphiphile, poly(2-methyl-2-oxazoline)-*b*-poly(2-phenyl-2-oxazine)-*b*-poly(2-methyl-2-oxazoline) (pMeOx-*b*-pPheOzi-*b*-pMeOx = A-pPheOzi-A), features the barely investigated aromatic PheOzi building block. In aqueous solution, at 40 °C, the polymer self-assembles into small (10–20 nm) and spherical polymer micelles, which transform to long interconnected worm-like aggregates upon cooling. At 32 °C, along with this cooling-induced order–order transition, the system solidifies with the viscosity increasing by several orders of magnitude. However, in stark contrast to before mentioned well-known inverse thermogelling systems, A-pPheOzi-A cannot form hydrogen bonds between repeat units and neither pMeOx nor pPheOzi homopolymers are thermoresponsive. Therefore, the question is: what is the mechanism of this order–order transition and concomitant inverse thermogelation?

**Figure 1:**
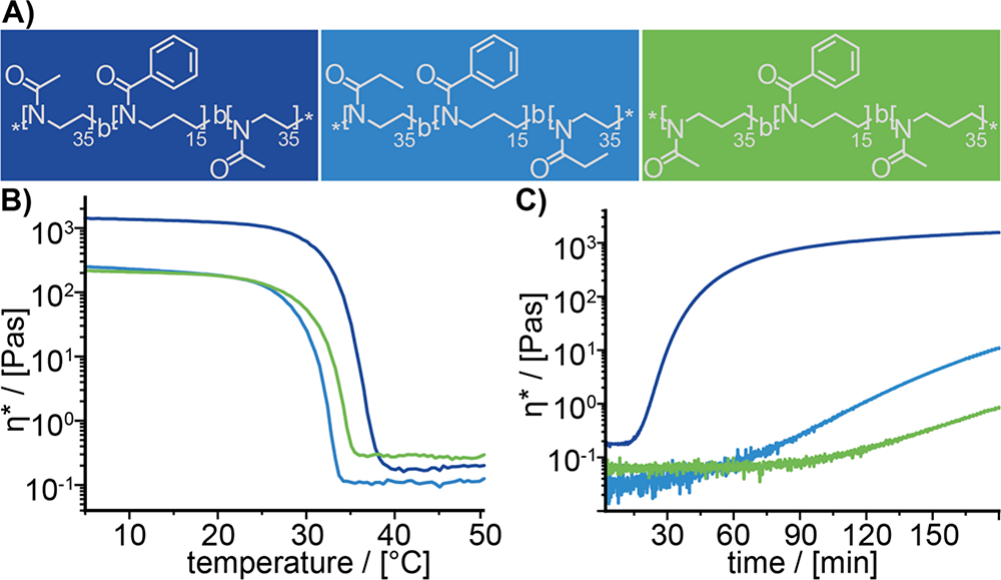
(A) Structure of the ABA type amphiphiles **P1–P3**. The herein introduced color code is used for all figures. (B) Gel properties of 15 wt % aqueous sol–gels of **P1** (dark blue), **P2** (light blue), and **P3** (green) at different temperatures. Complex viscosity η* from 5 to 50 °C (heat rate: 0.05 °C/s). (C) Gelation kinetics for 180 min. The gelation process was monitored at 5 °C by adding liquid samples at *t* = 0 min.

Using a selection of custom-synthesized derivatives, a wide variety of state-of-the-art analytic tools and molecular dynamics modeling, we could elucidate the molecular origins of this gelation mechanism in detail. This study not only reveals an alternative kind of polymer–polymer interactions responsible for this unusual order–order transition but also provides opportunities on how to precisely tailor the specific properties of the thermogels. This, in turn, is the fundamental requirement for the (future) use as smart biomaterial.

## Results and Discussion

Previously, we described the inverse thermogelling amphiphilic block copolymer A-pPheOzi-A (**P1**).^22^ As evidenced by cryogenic transmission electron microscopy (cryoTEM) investigations, **P1** forms spherical micelles at 40 °C in aqueous media that reversibly transform into worm-like micelles upon cooling. Interestingly, small structural changes in the hydrophobic core to e.g. 2-phenyl-2-oxazoline prevent both worm formation and thermogelation, which led to the assumption that hydrogel formation is based on the emergence of the worm-like micelles. However, cryoTEM images were recorded at dilute, nongelling concentrations. Here, to confirm that the order–order transition also occurs at gelling concentrations (*c*_gel_ > 5 wt %), we first conducted temperature dependent small-angle X-ray scattering (SAXS) at gel concentration (10 wt %) on **P1**. By evaluating the slopes of the scattering curves at different *Q* regions, the coexistence of spherical micelles and worm-like micelles in the gel state could be verified, with the latter disappearing in the sol state (Figure S1.1A). The qualitative analysis of the SAXS scattering^22,23^ profiles as well as additional wide-angle X-ray scattering (WAXS) data (Figure S1.1B) are summarized in the Supporting Information (SI) (Chapter S1).

While the use of PheOzi as the hydrophobic monomer has been shown to be essential for the order–order transition and thus the inverse thermogelation to occur, the influence of the hydrophilic blocks was yet unknown. Therefore, two closely related ABA type block copolymers with different A-blocks were synthesized. An additional methylene unit was added either to the side-chain of each hydrophilic repeat unit to obtain poly(2-ethyl-2-oxazoline)-*b*-poly(2-phenyl-2-oxazine)-*b*-poly(2-ethyl-2-oxazoline) (**P2**: pEtOx-*b*-pPheOzi-*b*-pEtOx) or to the backbone of each hydrophilic repeat unit yielding poly(2-methyl-2-oxazine)-*b*-poly(2-phenyl-2-oxazine)-*b*-poly(2-methyl-2-oxazine) (**P3**: pMeOzi-*b*-pPheOzi-*b*-pMeOzi) (Figure 1A, Chapter S2).

Similar to **P1**, both polymers **P2** and **P3** undergo inverse thermogelation, as well (Figure S2.1) and form worm-like micelles in aqueous media upon cooling as indicated by TEM imaging (Figure S2.2). However, significant differences in macroscopic gel properties of **P1**–**P3** were revealed by rheological measurements. Gel strength and persistence were tested in a temperature ramp experiment of **P1**–**P3** in the gel state, while the gelation kinetic was investigated in a time-sweep measurement of preheated polymer solutions (Figure 1B,C). In all three “disciplines” (gel strength, persistence, and gelation kinetics), **P1** showed the best performance whereas **P2** and **P3** exhibited a similarly lower gel strength and persistence combined with a slower gelation. Thus, it appears that the macroscopic properties of the resulting gel can be varied by changing the hydrophilic polymer block, while the basic mechanism of invers thermogelation accompanied by the unusual cooling-induced sphere-to-worm transition seemingly remains unaltered. This inevitably raises the question what the mechanism for this unusual order–order transition is and why or how the hydrophilic groups influence the cooling-induced gelation.

**Figure 2:**
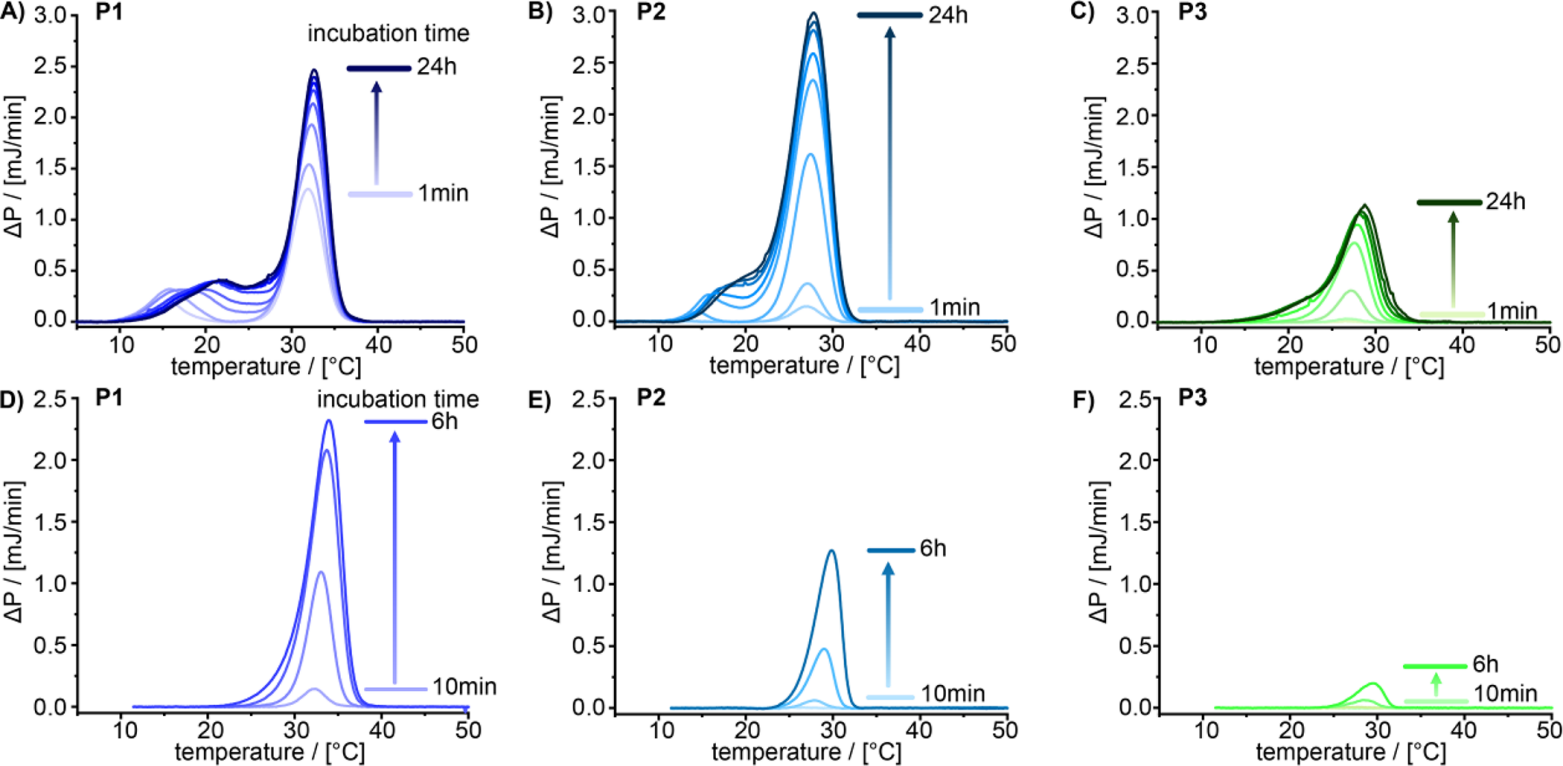
Micro-DSC thermograms of 1 wt % solutions of (A and D) **P1**, (B and E) **P2**, and (C and F) **P3**. Solutions where held at 2 °C (A–C) and 10 °C (D–F) for the time indicated prior to the thermoscan.

Micro-differential scanning calorimetric (Micro-DSC) was employed to gain first insights into the thermodynamic features of the order–order transition. Thermograms were obtained after equilibrating aqueous polymer samples at 2 °C (Figure 2A–C) or 10 °C, respectively (Figure 2D–F) for different incubation periods. For all polymers, the most prominent peak, which is associated with the order–order transition, coincides with the sol–gel transition. The signal intensity and, to a lesser extent, peak temperature of this signal evolves with increasing equilibrating time, suggesting some kind of maturation effect. Interestingly, for **P1** and **P2**, but not for **P3**, an additional small peak is visible in the thermograms prior to the main peak, which shifts toward the main peak.

At this point, we do not know the origin of this second, weaker peak. Surprisingly, if the samples are cooled to 10 °C instead of 2 °C, this secondary transition at lower temperature is not observed at all. We hypothesize that the variations in the thermograms might be related to dissimilarities in self-assemblies, although no differences could be discerned by cryoTEM in dependence of the incubation time.

**Figure 3:**
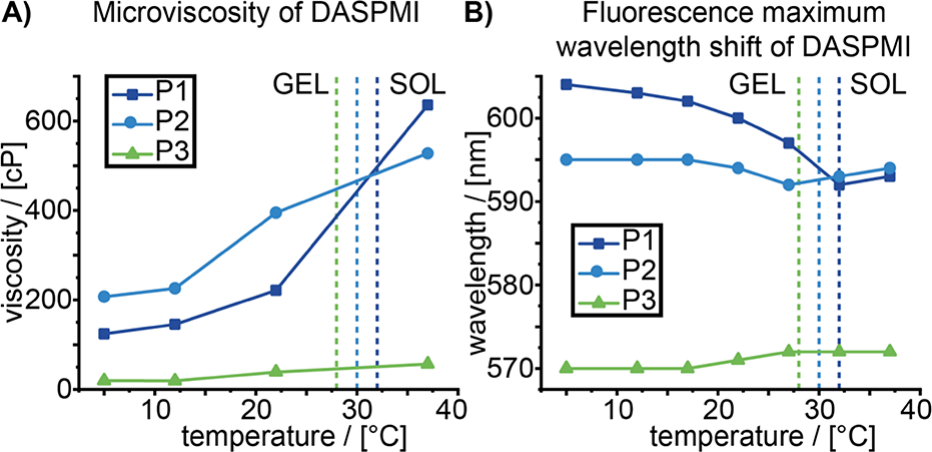
Microviscosity and fluorescence maximum wavelength shifts of DASPMI in 20 wt % solutions of **P1**, **P2**, and **P3** as a function of temperature. The dashed lines correspond to the respective sol–gel transition temperatures. (A) Microviscosity values for the microenvironment of DASPMI molecular rotors obtained from fluorescence lifetime experiments. (B) Fluorescence maximum wavelength shift of 5 μM DASPMI. Lines between data points are given only as guides for the eye.

Fluorescence spectroscopy can be used to uncover further properties of these self-assembled materials. In particular, molecular rotors such as 4,4′-difluoro-4-bora-3a,4a-diaza-*s*-indacene meso-substituted with para-dodecylphenyl, BODIPY-C12 (BPC12), and 2-(4-(dimethylamino)styryl)-1-methylpyridinium iodide (DASPMI)^24^ can probe changes in the mobility and polarity of their microenvironment with the change in self-assembly.^25^ Here, time-resolved and steady-state fluorescence measurements of the amphiphilic/hydrophilic DASPMI show a transition to a more polar (bathochromic shift) and (surprisingly) less viscous microenvironment in the gel state for **P1**, suggesting that the gelation causes a probe migration out of the condensed polymeric assembly closer to the polymer–water interface (Figure 3).

This trend is less pronounced for **P2** and not observable for **P3**. Although only indirect evidence, this indicates a stronger involvement of **P1**’s hydrophilic block in the gelation process and correlates with the higher macroscopic gel strength of **P1**. A more detailed discussion of the fluorescence maximum wavelength and lifetime changes of DASPMI as well as of BPC12 can be found in the SI (Chapter S3).

To gain more insights into the molecular interactions involved in the order–order transition, we conducted Raman spectroscopy. The Raman spectra for the hydrogels (20 wt %, 5 °C) and polymer sols (20 wt %, 40 °C) are quite similar (Figure S4.1A), but some distinct changes hint at differences in the polymer–polymer interactions between sol and gel state. At 1462–1464 cm^–1^, a small but clearly distinguishable peak is exclusively observed in the gels of **P1** and **P3** featuring methyl side chains. Unfortunately, both aromatic ring vibrations as well as CH_3_ and CH_2_ deformation vibrations ubiquitous in the polymer backbone and hydrophilic side chain appear in this region, making an unambiguous assignment challenging.

The amide band at 1601–1608 cm^–1^ does not shift for either polymer, but the full width-at-half-maximum decreases rather considerably for **P1** and **P2** from sol to gel (the signal-to-noise for **P3** was too poor to make a confident assessment). Similarly, a minor peak at around 1580 cm^–1^, attributed to the phenyl moiety shows increased intensity in **P1** in the gel state but not in **P2** and **P3**. Albeit in a different system (graphene interaction with polystyrene), such a shift has been attributed to electron donation into the aromatic system as observed in π–π stacking.^26^ These observations support the observations made in WAXS for **P1**, where a narrower peak also indicates better defined molecular interactions. In addition, we observed some notable differences in the fingerprint region and the OH region, which are discussed in the SI (Chapter S4).

**Figure 4:**
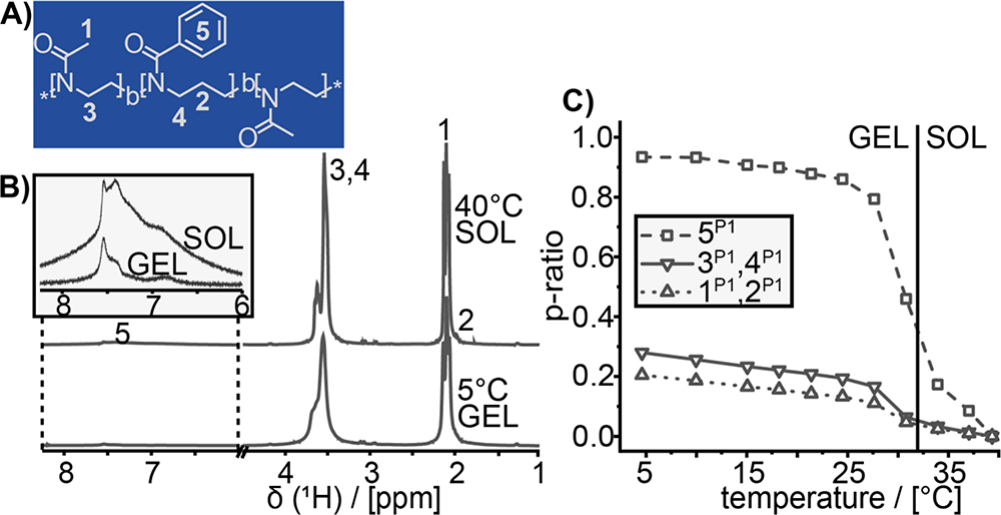
(A) Structure of **P1** including numbering scheme and (B) ^1^H NMR spectra at 5 and 40 °C of a 20 wt % sample in D_2_O. (C) p-ratios for the different proton peak integrals of **P1** as a function of temperature.

To this point, we have identified thermodynamic and spectroscopic cues that link differences in the molecular interactions with the macroscopic gel formation. But, the nature of the underlying interaction responsible for the order–order transition remains still unclear. Here, NMR spectroscopy gives insights into inter- and intramolecular interactions as well as details on mobility and spatial proximity.^27^ In a first step, ^1^H NMR spectra of the three polymers **P1**, **P2**, and **P3** were measured in aqueous solution at temperatures ranging from 5 to 40 °C. From the integrated peaks, we calculated p-ratios (Equation S2) as a temperature-normalized measure of signal loss. A p-ratio higher than zero quantifies the fraction of signal, which is lost into a broad background, as a result of dynamic slow-down throughout the sol–gel transition.^28^ Therefore, a high p-ratio correlates with a significant decrease in mobility of this functional group. All observed peaks were assigned to the respective protons (Figure 4B and Figures S5.1 and S5.2). Upon liquefaction, an increasing intensity along with a reduced line width and a more distinct fine structure can be observed for all signals, while the respective chemical shifts remain unchanged. The p-ratios of **P1** (Figure 4C) resemble those of **P2** and **P3** (Figures S5.1 and S5.2). The phenyl protons yield the highest p-ratios upon gel-formation with a maximum value of >0.9 for polymers **P1** and **P2**, whereas the analogous protons 5^**P3**^ experience a less pronounced intensity loss of 0.68.

Particularly, 5^**P1**^ shows a steep increase of p-ratios from 40 to 30 °C before reaching a plateau, while for **P2** and **P3**, a more gradual increase is observed. All other protons are less affected by the sol–gel transition (p-ratios <0.3). Overall, the hydrophilic block and the polymer backbone appear less affected by the transition, corroborating results by Weberskirch *et al*. and Černoch *et al*. describing thermoresponsive pOx-based homo- and copolymers.^28,29^

**Figure 5:**
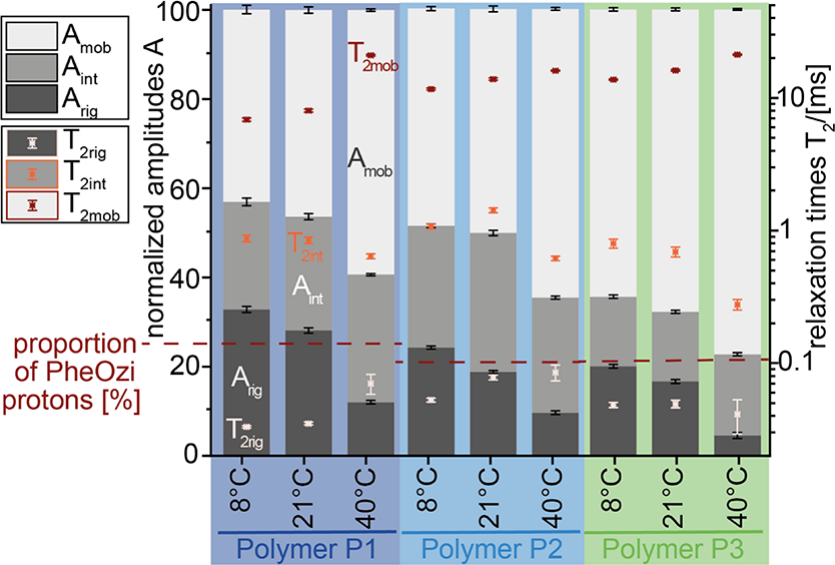
Low-field NMR data yielding three spin–spin relaxation times *T*_2rig_ (red cross), *T*_2int_ (orange cross), and *T*_2mob_ (light red cross) and the corresponding normalized amplitudes *A*_rig_ (dark gray bar), *A*_int_ (gray bar), and *A*_mob_ (light gray bar) of **P1**, **P2**, and **P3** at 8, 21, and 40 °C. The percentage of hydrophobic PheOzi protons of the three polymers is indicated by a horizontal red dotted line.

In ^1^H–^1^H NOESY measurements, an additional signal between aromatic and hydrophilic units indicates their proximity in the gel state for **P1** and **P2** (Figure S5.3) but not for **P3**. However, the reduced signal intensity indicated by the p-ratios shows that, with solution-state NMR, we are only monitoring a subensemble of relatively mobile polymer protons, which are still capable of fast segmental motions. As the micelles exceed the size of 10 nm, the observable NMR signals are not due to an overall fast tumbling but because of residual segmental mobility. To overcome this blind spot, low-field *T*_2_ relaxation measurements were performed to provide a quantitative view on the dynamic states of all protons (Figure 5). Important to note, in polymeric systems, *T*_2_ is mostly governed by residual dipolar couplings arising from the nonisotropic chain motion.^30^ In general, the shorter the *T*_2_ relaxation, the lower the mobility of the respective protons. The recorded signal decay curve results from the relaxation times of all protons in the sample. In total, four different relaxation times can be obtained by fitting this curve. After subtracting a highly mobile phase corresponding to bulk water, a rigid, an intermediate and a mobile polymeric phase can be distinguished (Figure 5). An exact description of the data evaluation including fit curves is shown in SI (Figure S6.1, Equations S3–S5).

Relaxation of the rigid phase is caused by local segmental mobility and rather short *T*_2__rig_ values below 90 μs are observed. Therefore, these signals cannot be resolved with high-resolution solution-state NMR measurements, which is in line with the course of the p-ratios. However, crystalline, glassy brush structures should have even lower *T*_2_ relaxation times of 15–25 μs. The somewhat higher value of *T*_2rig_ is most likely due to local motions like chain rotations or restricted segmental fluctuations, which average out the strong dipole–dipole couplings.^31^ Counterbalancing the rigid phase, the mobile phase signal fraction is decreasing with decreasing temperature, while the intermediate phase fraction remains rather constant. Due to the hydrophobic nature of the pPheOzi midblock, it is assumed to form the more rigid micellar core, whereas the hydrophilic A-blocks act as a corona around it. However, it is important to point out, that the proportion of rigid protons of hydrogel **P1** clearly exceeds the amount of pPheOzi protons. For hydrogel **P2**, only a slight excess and for hydrogel **P3** no excess of rigid phase can be observed. Consequently, both the hydrophilic pMeOx (**P1**) and—to a lesser extent—pEtOx (**P2**) blocks must therefore be involved in the formation of the more rigid phase-separated structure connected to hydrogel formation. Altogether, this suggests a structural rearrangement resulting in the formation of more condensed worm-like structures and thereby to gel formation. Additional low-field double-quantum measurements sensitive to constrained chain motions suggest a bottle-brush like appearance of the hydrogel’s outer shell (Figures S6.2–S6.4), which relates to the mobile and intermediate moieties and thus complements relaxation data.

**Figure 6:**
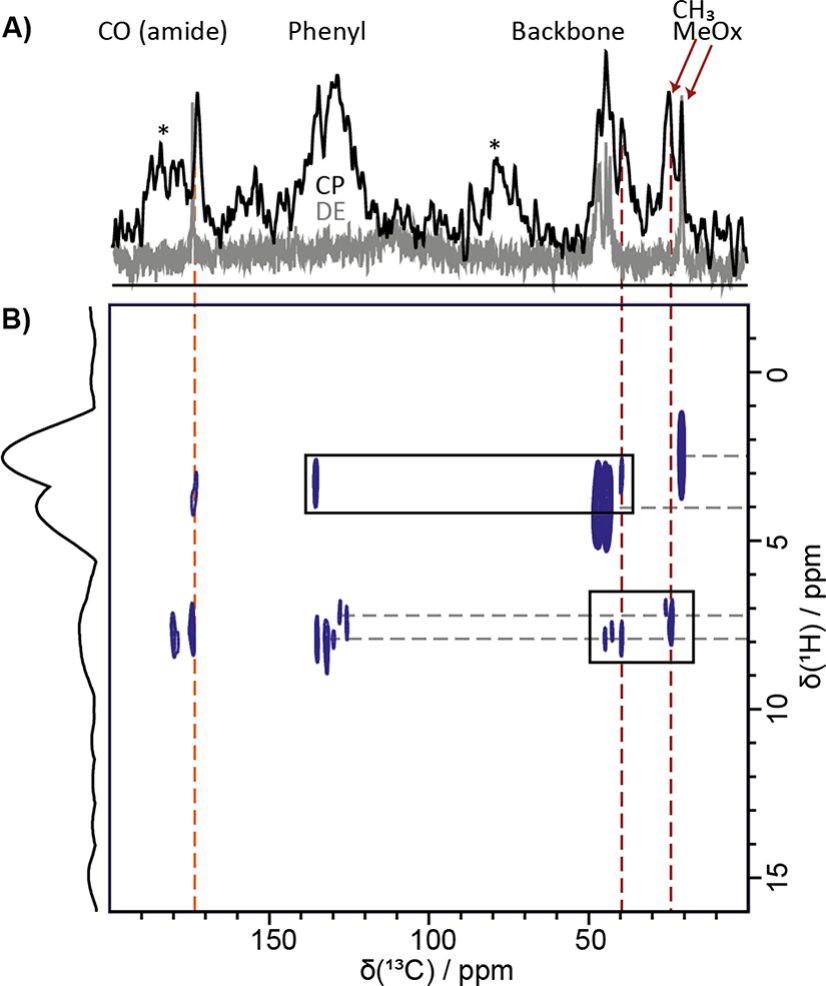
Solid-state NMR spectra of a 20 wt % **P1** sample. (A) Overlay of the ^13^C solid-state NMR spectra recorded at 9.4 T and 5.3 kHz MAS using DE and short interscan delay of 1 s (gray) or CP MAS with 2 ms contact time (black). Spinning sidebands are indicated by asterisks. (B) ^1^H–^13^C FSLG HETCOR MAS spectrum recorded at 9.4 T and a MAS rate of 5 kHz using a contact time of 2 ms. 122 t_1_ FID increments were acquired using a recycle delay of 2 s, each with 240 coadded transients. Direct CH contacts are indicated by dotted gray lines.

Unlike ^1^H NMR spectra in solution, solid-state ^13^C cross-polarization (CP) and ^13^C direct excitation (DE) spectra can resolve both rigid and more mobile carbon atoms within one sample. Here, the most significant revelation from the ^13^C CP MAS experiments of hydrogel **P1** is the presence of two different species of methyl side chains, one more mobile and one more rigid (Figure 6, red arrows). Importantly, only the rigid environment exhibits close proximity to the phenyl groups as indicated by a cross-peak in the ^1^H–^13^C FSLG HETCOR spectrum (Figure 6).

Identical measurements were performed on hydrogels **P2** and **P3** with only one mobile methyl group (**P3**) or ethyl group (**P2**) moiety being observable (Figure S7). Overall, experimental data suggest an increased interaction between the methyl group of the hydrophilic MeOx units and the hydrophobic PheOzi block (**P1**) at a lower temperature. Domains of lower mobility are formed with the involvement of the hydrophilic MeOx units, which is reflected by the rigid component of the *T*_2_ relaxation times. This explains the experimentally observed critical involvement of the hydrophilic polymer blocks in the order–order transition and formation of the hydrogel. Interestingly, this means that the interaction is favored over hydrophilic–hydrophobic phase separation in aqueous environments. The variation of only these hydrophilic outer blocks (**P2** and **P3**) directly influences the macroscopic gel strength decreasing from **P1** > **P2** > **P3**.

**Figure 7:**
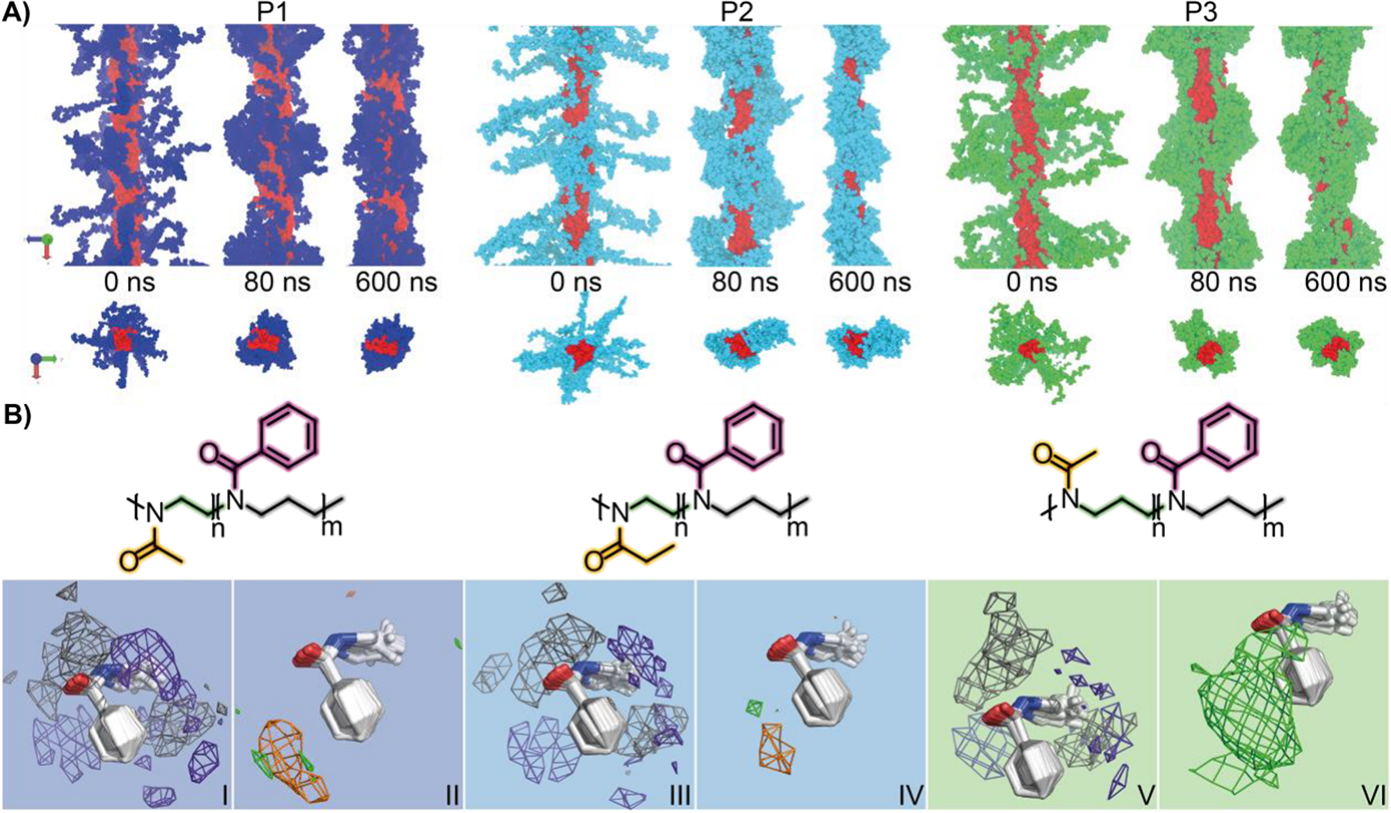
Results of molecular modeling of worm-like micelles comprising **P1** (left), **P2** (middle), or **P3** (right). (A) Simulation snapshots from two angles (top, side view and bottom, top view), showing pPheOzi monomers as red and A-blocks as blue/turquoise/green VDW spheres. The simulation box and about half of each neighboring image along the Z-axis are illustrated without solvent molecules. (B) Occupancy density analyses around aligned pPheOzi residues (white sticks), showing hotspots for different polymer structures as meshes. In B^I^, B^III^, and B^V^, the violet densities represent pPheOzi side chains and the gray densities are pPheOzi backbone atoms (isovalues: 0.08). Structures in B^II^, B^IV^, and B^VI^ depict densities (isovalues: 0.03) for A-block backbone atoms (green) and side chain atoms (orange).

To further dissect the interplay between different polymer moieties, we performed all-atom molecular dynamics (MD) simulations of single worm-like micelles with full length **P1**, **P2**, or **P3** amphiphiles at 5 °C. We modeled the pPheOzi blocks as a central inner strand, which is surrounded by the corresponding A-blocks (pMeOx, pEtOx, pMeOzi) stretching out into the solvent (water). Throughout the 600 ns long simulations, a single worm-like strand of pPheOzi blocks is preserved in all cases. More interestingly, however, the peripheral A-block repeat units approach the initially solvent-exposed hydrophobic repeat units while partially shedding their hydration sphere, clearly corroborating our model of a hydrophilic shell condensing onto the hydrophobic core (Figure 7A).

**Figure 8:**
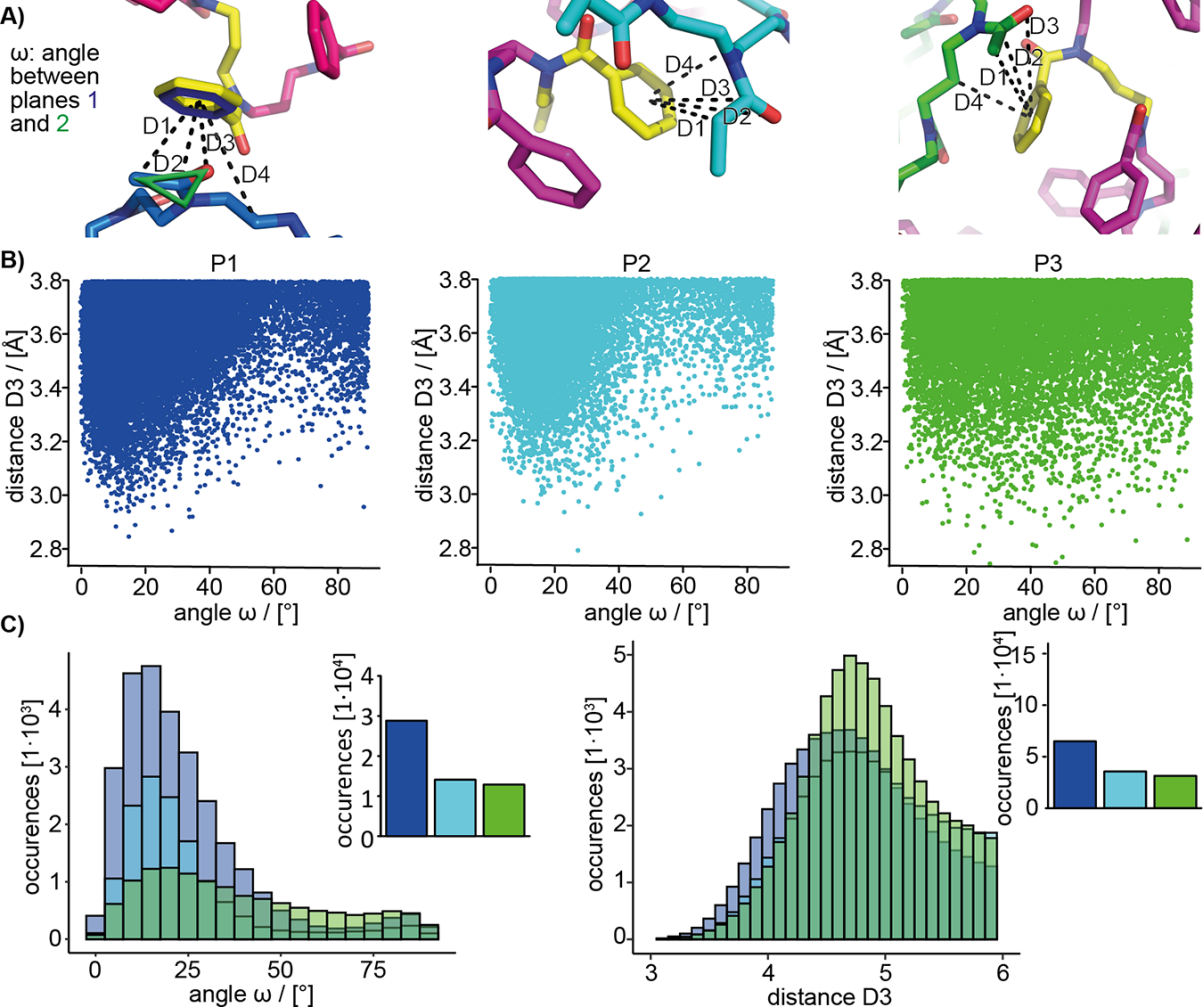
(A) Illustrations describing relevant distances and the angle ω. )B) 2D plots for distance D3 and the respective angle ω. (C) Histogram (bin size: 5°) for ω for all A-block carbonyl oxygen atoms, which are located ≤3.8 Å to the phenyl ring centroid (corresponding to distance D3), and histograms (bin size: 0.1 Å) for distance D3 up to 6 Å between pPheOzi moieties and the nearest polymer atoms (excluding hydrogen atoms). Plots show the total amount of occurrences for all snapshots of all 104 pPheOzi residues used for density calculation. Barplots next to each histogram further compare the total amount of occurrences below 4 Å for each polymer type.

Consequently, radii of gyration of all self-assemblies decrease quickly and reach a narrow fluctuation range, with pMeOzi showing higher values due to its longer backbone (Figure S8.1B). The amount of water molecules within 5 Å around polymer residues also decreases quickly, especially for A-block monomers (Figure S8.1C). While the overall structures become more compact, not all A-blocks come into close contact with pPheOzi residues *in silico*, which in turn are also not completely shielded from the solvent at the end of the simulations, corroborating the observations made in solution NMR spectroscopy. Solvent-exposed pPheOzi blocks could be interpreted as sticky patches, which help to mechanically connect different worm-like micelles, adding to the high storage modulus of the gels.^22^ The evidence of such sticky contacts in worm-like micelles was recently discussed by Thurn and Hoffmann.^32^ However, we cannot completely rule out that these patches result (in part) from an insufficient number of polymers in our model, as the exact composition of the micelle was not available as *a priori* input from experimental data. *In silico*, about 53% of all pMeOx, 62% of all pEtOx, and 83% of all pMeOzi repeat units keep a minimum average distance of more than 5 Å to the pPheOzi blocks over the last 100 ns (including hydrogen atoms for calculation). These values correspond to 40%, 49%, and 66% of all protons and, thus, agree well with the amount of mobile protons found in low-field relaxation time measurements (43%, 49%, and 64%) (Figure 5). For **P1**, these MeOx groups can be connected to the methyl group signal, that showed no cross peak with the aromatic rings of pPheOzi in the ^1^H–^13^C HETCOR spectrum. Notably and surprisingly, despite their larger sizes and less hydrophilic nature compared to pMeOx, pEtOx and pMeOzi do not lead to a greater coverage of pPheOzi blocks (Figure S8.1D) but instead form larger clusters on their own (compare last snapshots in Figure 7A). This *in silico* observation mirrors the macroscopic gel strength. To take a closer look into the self-assemblies, the average occupancy densities of polymer moieties around pPheOzi moieties were analyzed (Figure 7B and Figure S8.2). Interestingly, pMeOx and, more specifically, its acetyl side chains are predominantly located close to the aromatic ring of pPheOzi near its carbonyl group. In contrast, adjacent aromatic rings can be found below or above the PheOzi side chain excluding the occupied volume of the pMeOx acetyl groups. The pPheOzi backbone atoms are mainly surrounded by other pPheOzi residues. Using the same density cutoff values for the simulation of **P2** shows a slightly reduced occupancy for A-block side chains around the pPheOzi residues. Notably, in case of **P3**, an A-block side chain density around the pPheOzi residues at this level cannot be detected. Instead, interactions with its backbone atoms are more pronounced. For the pPheOzi aromatic residues analyzed during density calculation, we further measured distances to the nearest polymer moieties, as well as the angle ω between the planes of the aromatic ring (Figure 8A, plane 1) and nearby amide groups (Figure 8A, plane 2).

Distances D1–D4 reflect the orange and green densities in Figure 7B. While overall considerable similarities between the different polymers are noticeable, the number of occurrences for distances D1–D3 below 4 Å is clearly highest in case of pMeOx (Figure 8C and Figure S8.3), highlighting more interactions between the acetyl side chains and the phenyl rings. Overall, distances show distributions similar to the results of our WAXS experiments, but MD allows for attribution to specific groups. The distance to the ring centroid is lowest for the pMeOx methyl group, while the backbone is situated further away. This supports hydrophobic interactions between aromatic groups of pPheOzi and the methyl side chain of pMeOx repeat units, again corroborating results from NMR spectroscopy. While the existence of specific interactions between lone pairs and aromatic systems is actively debated^33^ and the ability of classical force fields for capturing these can certainly be questioned,^34^ occurrences of an angle ω ≤ 90° in combination with a distance lower than 3.8 Å for a carbonyl oxygen to an aromatic ring centroid were previously suggested for n_Am_ → π*_Ar_ or π_Am_···π_Ar_ interactions, *e.g*., in peptoids.^35,36^ Indeed, very low values for ω with a median of 19° for the above-mentioned distance cutoff were retrieved for **P1** and **P2** (Figure 8B,C), although the occurrences within 3.8 Å are much more numerous for **P1**. In contrast, much fewer coplanar side chain orientations were observed for **P3** (median value: 33°). Instead, in accordance with density hotspots, low values of distance D4 between the A-block backbones and the aromatic rings are more common (Figure S8.3). In summary, the strongest indication for potential n_Am_ → π*_Ar_ or π_Am_···π_Ar_ interactions is found for **P1**, which directly correlates with its macroscopic gel properties. Counterintuitively, **P2** and **P3** having less hydrophilic A-blocks show a lower probability of interaction between the hydrophilic A and hydrophobic PheOzi blocks. Noncovalent interactions between different blocks of block copolymers are of course known, especially that they can affect the self-assembly. Established examples for such interactions are H-bonding or ionic interactions, including recently reported interactions between PEG and a cationically charged hydrophobic block.^37^ However, we are unaware of a similar interaction between two nonionic, non-H-bonding capable polymer blocks as elucidated here to have been previously reported. Importantly, it should be noted that the force fields available for MD simulation are inherently incapable to provide quantum-chemistry level insights such as the suggested n_Am_ → π*_Ar_ or π_Am_···π_Ar_ interactions. For this, quantum mechanical modeling such as density-functional theory calculations would be needed. We will attempt this in future work, but we expect that it will be nontrivial to accurately describe such relatively weak interactions in a complex system such as ours.

In a broader context, interactions between tertiary amides of pOx and phenyl groups as reported here could be very relevant for interactions of pOx, pOzi, poly(vinylpyrrolidone), poly(*N*,*N*-dimethyl methacrylate) or polypeptoids such as polysarcosine (all featuring tertiary amides in every repeat unit) with other biologically relevant macromolecules comprising aromatic moieties, in particular peptides, proteins, or antibodies. In fact, recent simulations of Interferon-α2a conjugated with pEtOx showed favorable interactions of the polymer with aromatic amino acids, in contrast to PEG, which preferred interacting with positively charged side chains.^38^ Such distinct differences in interactions of hydrophilic stealth polymers could also be very relevant in drug-loaded polymer micelles, polymer–drug conjugates, liposomes, antibody–polymer drug conjugates, and lipid nanoparticles, in the latter especially for the interaction with cationic lipids. Advanced NMR spectroscopy, including ssNMR employing domain editing *via T*_1_-relaxation time filters, has been previously used in combination with quantum-chemical calculations to unravel another unusual interaction within active pharmaceutical ingredient clusters being part of organogels found within the pores of mesoporous silica microparticles. Although very different structures are discussed in this work by Brus *et al*., the suggested −OH → π_Ar_ interactions could be viewed as nonclassical interactions in nonclassical gel phases similar to the interactions suggested in our current contribution.^39^

In summary, the MD simulations conclusively support our extensive analytical data, in particular by NMR spectroscopy, regarding the postulated mechanism of the hydrophilic pMeOx (and to a lesser extent, pEtOx and pMeOzi) interaction with the hydrophobic repeat units as the underlying cause for the observed and highly unusual order–order transition. Critical in this context is the notable condensation of the hydrophilic corona, which is needed for the sphere-to-worm transition upon cooling. The less pronounced interactions of hydrophilic A-blocks found *in silico* excellently corroborate our finding of more mobile groups for **P2** and **P3**, as determined by NMR spectroscopy and experimentally determined differences in rheological properties, *i*.*e*., much weaker hydrogels for **P2** and **P3**. This interaction discovered here could potentially become more widely usable as control principle for polymer self-assembly, for example, by employing *N*,*N*-dimethyl methacrylate as polymer building blocks. Also, one can hypothesize that further fine-tuning of the gel properties should be possible by combining pMeOx, pEtOx, or other building blocks in different ratios in the hydrophilic block or by modifying the electron density of the aromatic ring by substitution. This contribution confirms also once more that polymer self-assembly can be controlled by much more complex factors than thought only a few years ago, which has been discussed recently in some detail for drug loaded micelles.^40−42^

## Conclusion

Using a wide selection of complementary analytical tools, a detailed picture of an unusual order–order transition in conjunction with an inverse thermogelation of aqueous solutions of a special group of amphiphilic block copolymers was obtained. Using micro-DSC, Raman, and fluorescence spectroscopy, a strong influence of the hydrophilic blocks on the gelation mechanism and thermodynamics was indicated, consistently reflecting the trend of decreasing gel strength, persistence, and a slower kinetic from **P1** to **P3**. Detailed NMR spectroscopic investigations revealed polymer–polymer interactions between the hydrophilic pMeOx blocks and the hydrophobic aromatic pPheOzi moieties of **P1** in the hydrogel state. A condensation of the hydrophilic corona upon gelation is observed by low-field NMR relaxation measurements and validated by MD simulations. However, more detailed quantum mechanical calculations will be needed to confirm the exact nature of this suggested interaction, as MD simulations are inherently incapable to provide this. Nevertheless, our results strongly suggest that a nonclassical interaction of the hydrophilic and hydrophobic repeat units seems to be the major driving force for the gelation with decreasing extent for **P1** to **P3** as indicated in the macroscopic gel properties. This is rather unusual, as the dehydration of (highly hydrophilic) MeOx units occurs in favor of inter- and intramolecular interactions with hydrophobic repeat units. We believe that the analysis of this self-assembly mechanism can be used to design stimulus responsive materials and will generally help to further improve our understanding of the complex interactions of polymers in solutions. For example, future work should include further variation of the hydrophilic blocks to PEG, which has been suggested to preferably interact with cations over aromatic systems or modification of the electron density in the aromatic ring by relevant substituents. In a broader context, the presented results will be of importance for much needed improved understanding of the interactions of polymeric biomaterials with biological systems, in particular peptides and proteins.

## Materials and Methods

A more detailed description of materials and the experimental methods is included in the Supporting Information.

### Polymer Synthesis

Polymer synthesis was performed as described previously for Me-pMeOx_35_-*b*-pPheOzi_15_-*b*-pMeOx_35_ (**P1**)^22^ to obtain Me-pEtOx_35_-*b*-pPheOzi_15_-*b*-pEtOx_35_-EIP (**P2**) and Me-pMeOzi_35_-*b*-pPheOzi_15_-*b*-pMeOzi_35_-PipBoc (**P3**).

### Gel Permeation Chromatography

Gel permeation chromatography (GPC) was performed on a Polymer Standard Service PSS (Mainz, Germany) system. Specifications: pump mod. 1260 infinity, MDS RI-detector mod. 1260 infinity (Agilent Technologies, Santa Clara, CA); precolumn, 50 × 8 mm PSS PFG linear M; 2 columns, 300 × 8 mm PSS PFG linear M (particle size 7 μm; pore size 0.1–1.000 kg/mol) with hexafluoroisopropanol (HFIP, containing 3 g/L potassium trifluoroacetate (KTFA)) as eluent calibrated with PEG standards (molar masses from 0.1 to 1000 kg/mol). The columns were held at 40 °C, and the flow rate was set to 0.7 mL/min. Dried polymer powders were dissolved in eluent and filtered through 0.2 μm PTFE filters (Rotilabo, Karlsruhe, Germany).

### Differential Scanning Calorimetry (DSC)

All measurements were performed using aluminum crucibles on a calibrated DS 204 F1 Phoenix system from NETZSCH (Selb, Germany) equipped with a CC200 F1 controller unit from −50 to 200 °C with three heating and two cooling phases and a cooling rate of 10 °C/min. The third heating cycle was used to analyze the glass transition temperature of dried polymer powders.

### Rheology

All experiments were performed using an Anton Paar (Ostfildern, Germany) Physica MCR 301 system utilizing a plate–plate geometry (25 mm diameter) equipped with a solvent trap and Peltier element for temperature adjustment. All aqueous 15 wt % samples were dissolved at room temperature stirring constantly and incubated at 5 °C for 48 h. In addition, pictures were taken to visualize the gels. A temperature-sweep was performed in oscillation mode from 5 to 50 °C (heating rate: 0.05 °C/s) using a fixed amplitude of 0.1% and angular frequency of 10 rad/s. The long-time gelation experiment at 5 °C was performed at an amplitude of 0.1% and an angular frequency of 10 rad/s for several hours.

### Transmission Electron Microscopy

For transmission electron microscopy (TEM) experiments, the polymers were dissolved in DI water to a final concentration of 20 g L^–1^ and stored at room temperature. Four hundred mesh copper–rhodium grids (maxtaform) with a homemade carbon layer were glow discharged in air for 1.5 min at medium power in a Harrick PDC-002 plasma cleaner. The 20 g L^–1^ sample was diluted (1/125 or 1/625), and 8 μL was incubated on the grids for 1 min before blotting (Whatman filter paper No. 50). The grids were washed with water (three times) and 2% w/v uranyl acetate (three times). After the last dose of uranyl acetate was applied, the grid was left to incubate for 5 min before blotting. A single-tilt room temperature holder in an FEI Tecnai G2 Spirit TWIN transmission electron microscope equipped with a tungsten emitter at 120 kV was used. Images were recorded with an Eagle CCD camera under low-dose conditions. The micrographs were binned two times resulting in a pixel size of 2.2 Å per pixel at specimen level.

### Small- and Wide-Angle X-ray Scattering (SAXS, WAXS)

SAXS and WAXS experiments were carried out using an in-house setup, which was built by Fraunhofer EZRT (Fürth, Germany). It consists of a MicroMax-007 HF X-ray source (Rigaku, Japan) and a Eiger R 1 M detector unit (Dectris, Switzerland). The sample–detector distance can be varied between 5 cm and 3.5 m, which corresponds to possible Q-values between 0.005 and 5 Å^–1^. The complete setup is operated in a vacuum below 0.1 mbar to reduce air scattering. The sample solutions were placed in quartz capillaries (inner diameter, 1 mm; wall thickness, 10 μm) (Hampton Research, Aliso Viejo, CA), which were positioned perpendicularly to the X-ray beam. The presented experiments were done at sample–detector distances of 57, 565, and 1560 mm with an integration time of 15 min for the shortest distance and 240 min for the two longer configurations. All distances were calibrated using a silver behenate standard sample. For each sample, data was acquired for different temperatures between 5 and 50 °C. To achieve thermal equilibrium, the sample (10 wt % aqueous solution) was kept at the desired temperature for 15 min prior to each measurement. The SAXS data, which was obtained at the two largest distances, was calibrated in terms of absolute intensities using glassy carbon as a secondary calibration standard.^43,44^ The scattering curves of the hydrogels were obtained by azimuthal integration taking the samples thickness, X-ray transmission, detector accuracy, setup geometry, and solvent scattering into account following the standard procedures described in the literature.^45^

### Micro-differential

Micro-differential scanning calorimetry measurements were conducted with a Malvern MicroCal PEAQ-DSC microcalorimeter. The heat of the sample was measured relative to pure water, and the enthalpy values were normalized to the molar concentration of the aromatic repeat units. After complete dissolution, the samples were stored in the refrigerator at 4 °C for about 48 h, degassed at 5 °C, transferred to the instrument precooled at 2 or 10 °C, and kept at the temperature for different times, as indicated, prior to heating. Each sample was heated with the rate of 1 °C/min to 100 °C, after which they were cooled again to the starting temperature with the same rate.

### Fluorescence Spectroscopy

2-(4-Dimethylamino)styryl)-1-methylpyridinium iodide (DASPMI) was purchased from Molecular Probes Inc., Life Technologies and used without further purification. 4,4′-Difluoro-4bora-3a,4a-diaza-*s*-indacene meso-substituted with para-dodecylphenyl moiety (BPC12) was synthesized in our group according to the literature methods.^46^ Gibco Dulbecco’s phosphate-buffered saline (DPBS) pH 7.25 was purchased from Thermo Fisher Scientific. Steady-state and time-resolved fluorescence of the molecular rotors DASPMI and BPC12 were obtained using a FLS-1000 spectrofluorometer (Edinburgh Instruments) equipped with a thermocontrolled cuvette holder. Fluorescence intensity decay curves were measured using time-correlated single photon counting (TCSPC) system (PicoQuant, GmBH) described earlier.^24^ Viscosities were calculated at 5, 12, 22, and 37 °C by eq S1.

### Raman Spectroscopy

The Raman spectra were recorded on an alpha 300R^+^ confocal Raman microscope from WITec GmbH (Ulm, Germany) equipped with a 50× objective (NA 0.8, Epiplan Neofluar, Zeiss, Germany) and a 532 nm laser (39.4 mW).

### Nuclear Magnetic Resonance (NMR) Experiments

All experiments in solution were performed on a Bruker Avance III HD 600 spectrometer (Karlsruhe, Germany) operating at 600.4 MHz equipped with a BBFO 5 mm probe using a BCU-02 temperature control unit. Low field NMR measurements were performed using a Bruker Minispec mq20 (Bruker, Karlsruhe, Germany) operating at 19.9 MHz with a 90° pulse length of 2.5 μs and a dead time of about 15 μs. Solid-state NMR (ssNMR) measurements were performed using a 4 mm double-channel Bruker probe at 9.4 T using between 3 and 7 kHz magic angle spinning (MAS).

### Molecular Modeling

Three systems, each containing eight chains of a single polymer type, were modeled. All modeling was performed with MOE (Molecular Operating Environment 2019.01).^47^ RESP partial charges^48^ of single monomers used as building blocks were derived from calculations with Gaussian 09 Rev. C.01^49^ (Hartree–Fock level of theory, 6-31G* basis set); parameters based on the Amber14ffSB^50^ and GAFF2^51^ force fields were assigned *via* antechamber and parmchk2 of AmberTools18.^52,53^ Simulations were performed using NAMD 2.13^54^ with 2 fs time steps. Analyses were performed using cpptraj.^55^ Average densities for polymer groups around pPheOzi monomers were retrieved as described in detail in the SI. Additionally, several distances between these pPheOzi monomers and the other polymer residues were analyzed, as well as the angle ω between the plane of nearby amide (N—(C=O)—C) groups and the phenyl ring plane.

## Supporting Information Available

Discussions of materials and methods, tables of summary of SAXS analysis for a **P1** hydrogel, characteristics of the polymers **P1**, **P2**, and **P3**, and calibration equations for BPC12, and figures of SAXS and WAXS scattering curves of aqueous solutions of **P1**, pictures of aqueous samples of **P2** and **P3**, TEM images of aqueous solutions of **P2** and **P3**, kinetic curves and corresponding calibration curves of DASPMI, fluorescence lifetimes of 5 μM DASPMI and 5 μM BPC12 in **P1**, **P2**, and **P3**, microviscosity values for the microenvironment of DASPMI and BPC12 molecular rotors in **P1**, **P2**, and **P3**, normalized fluorescence spectra of 5 μM DASPMI and 5 μM BPC12 in **P1**, **P2**, and **P3**, temperature dependent Raman experiments of **P1**, **P2**, and **P3**, structures of **P2** and **P3** including numbering scheme and ^1^H NMR spectra and p-ratios, ^1^H–^1^H NOESY NMR experiments of **P1**, FID, MSE, and HE decays of **P1**, **P2**, and **P3**, DQ NMR build-up and decay measured of **P1**, **P2**, and **P3**, ^13^C DE and CP MAS NMR spectra and ^1^H–^13^C HETCOR MAS spectra of **P1**, **P2**, and **P3**, illustration of MD starting structures, and further *in silico* analyses (radii of gyration, hydration, contact areas, distance histograms) (PDF)
