# Supplementary material for: Unraveling an Alternative Mechanism in Polymer Self-Assemblies: An Order–Order Transition with Unusual Molecular Interactions between Hydrophilic and Hydrophobic Polymer Blocks
Source: ACS Nano. 2023 Mar 27;17(7):6932–42. doi: 10.1021/acsnano.3c00722 (PMC10100562; doi:10.1021/acsnano.3c00722)
Supplement: Supplementary file 1 — nn3c00722_si_001.pdf [file nn3c00722_si_001.pdf]

Supporting Information

Unravelling an Alternative Mechanism in Polymer Self-Assemblies: An Order-Order Transition with Unusual Molecular Interactions between Hydrophilic and Hydrophobic Polymer Blocks

Lukas Hahn[a,b§], Theresa Zorn[c§], Josef Kehrein[d#], Tobias Kielholz[e], Anna-Lena Ziegler[a], Stefan Forster[a], Benedikt Sochor[f$], Ekaterina S. Lisitsyna[g], Nikita A. Durandin[g], Timo Laaksonen[g,h], Vladimir Aseyev[i], Christoph Sotriffer[d], Kay Saalwächter[j], Maike Windbergs[e], Ann-Christin Pöppler[c], and Robert Luxenhofer[a,i]**

[a]Institute for Functional Materials and Biofabrication, Department of Chemistry and Pharmacy, Julius-Maximilians-University Würzburg, Röntgenring 11, 97070 Würzburg, Germany

[b]Institute of Pharmacy and Food Chemistry, Department of Chemistry and Pharmacy, Julius-Maximilians-University Würzburg, Am Hubland, 97074 Würzburg, Germany

[c]Center for Nanosystems Chemistry & Institute of Organic Chemistry, Department of Chemistry and Pharmacy, Julius-Maximilians-University Würzburg, Am Hubland, 97074 Würzburg, Germany

[d]Institute of Pharmacy and Food Chemistry, Department of Chemistry and Pharmacy, Julius-Maximilians-University Würzburg, Am Hubland, 97074 Würzburg, Germany

[e]Institute of Pharmaceutical Technology and Buchmann Institute for Molecular Life Sciences, Goethe University Frankfurt, Max-von-Laue-Str. 9, 60438 Frankfurt am Main, Germany

[f]Chair for X-Ray Microscopy, Julius-Maximilians-University Würzburg, Josef-Martin-Weg 63, 97074 Würzburg, Germany

[g]Faculty of Engineering and Natural Science, Tampere University, Korkeakoulunkatu 8, 33720 Tampere, Finland

[h]Division of Pharmaceutical Biosciences, Faculty of Pharmacy, University of Helsinki, Viikinkaari 5 E, 00014 Helsinki, Finland

[i]Soft Matter Chemistry, Department of Chemistry, Helsinki Institute of Sustainability Science, Faculty of Science, University of Helsinki, 00014 Helsinki, Finland

[j]Institute of Physics-NMR, Martin-Luther-Universität Halle-Wittenberg, Betty-Heimann-Str. 7, 06120 Halle, Germany

*Email: robert.luxenhofer@helsinki.fi

*Email: ann-christin.poeppler@uni-wuerzburg.de

[§]L.H. and T.Z. contributed equally to this paper

Polymer synthesis 3
Gel permeation chromatography 4
Differential Scanning Calorimetry (DSC) 4
Rheology 4
Transmission electron microscopy 4
Small- and wide-angle X-ray scattering (SAXS, WAXS) 5
Micro calorimetry (µCAL) 5
Fluorescence spectroscopy 6
Raman spectroscopy 7
Nuclear magnetic resonance (NMR) experiments in solution 7
Low field nuclear magnetic resonance 8
Solid-state nuclear magnetic resonance 8
Molecular modeling 8
Complementary Material 10
Chapter S1 – SAXS and WAXS measurements 10
Chapter S2 – Polymer characteristics 12
Chapter S3 – Fluorescence spectroscopy of molecular rotors 13
Chapter S4 – Raman measurements 18
Chapter S5 – NMR experiments in solution 20
Chapter S6 – Low-field NMR experiments at 20 MHz 22
Chapter S7 – Solid-state NMR experiments 29
Chapter S8 – Molecular modeling 30
Author Contributions 36

Materials and Methods

All chemicals and reagents were used from Merck (Steinheim, Germany) or TCI-chemicals (Eschborn, Germany) and were used as received unless otherwise mentioned. The polymer pMeOx-*b*-pPheOzi-*b*-pMeOx (= A-pPheOzi-A) was prepared and used as described previously.[22] The monomers 2-ethyl-2-oxazoline (EtOx) and 2-methyl-2-oxazine (MeOzi) were synthesized as described by Witte and Seeliger.[1] Deuterated dichloromethane (d_2DCM) and D_2O as NMR solvent were obtained from Deutero GmbH (Kastellaun, Germany). Methyl trifluoromethylsulfonate (MeOTf), EtOx, MeOzi and PheOzi were refluxed over CaH_2 for several hours and distilled under reduced pressure. Benzonitrile (PhCN) was dried over phosphorus pentoxide.

Polymer synthesis

Briefly, the initiator MeOTf (1 eq.) was added to a dried and argon flushed flask and dissolved in the 21 mL of solvent (PhCN). The first monomer EtOx or MeOzi (35 eq.) was added to the reaction mixture and heated to 100°C for several hours. After complete consumption (verified *via* ^{1}H NMR), the monomer for the second block PheOzi (15 eq.) was added at room temperature. The reaction mixture was heated to 120°C over night. After complete monomer consumption was confirmed, the 3rd block EtOx or MeOzi (35 eq.) was added and stirred for 2 several hours at 100°C. Termination was carried out by the addition of 3 eq. of 1-Boc-piperazine (PipBoc) or Ethylisonipecotate (EIP) at 50°C and kept on stirring for 6 hours. The solvent was removed at reduced pressure. The raw product was dissolved in deionized (DI) water and dialyzed (MWCO 1 kDa, cellulose acetate) against DI water for 3 days. The polymer solution was lyophilized, and the polymers were obtained as a white powder.

Me-pEtOx$_{35}$-b-pPheOzi$_{15}$-b-pEtOx$_{35}$-EIP:

Initiation:	MeOTf	176 mg	(1.07 mmol; 1 eq)
1. Block:	EtOx	3.63 g	(36.62 mmol; 34 eq)
2. Block:	PheOzi	2.58 g	(16.0 mmol; 15 eq)
3. Block:	EtOx	3.64 g	(36.72 mmol; 34 eq)
Termination:	EIP	495 mg	(3.15 mmol; 3 eq)
Yield:		8.58 g (82.2%)	

Me-pMeOzi$_{35}$-b-pPheOzi$_{15}$-b-pMeOzi$_{35}$-PipBoc:

Initiation:	MeOTf	128 mg	(0.78 mmol; 1 eq)
1. Block:	MeOzi	2.52 g	(25.42 mmol; 33 eq)
2. Block:	PheOzi	1.83 g	(11.35 mmol; 15 eq)
3. Block:	MeOzi	2.54 g	(25.62 mmol; 33 eq)
Termination:	PipBoc	409 mg	(2.19 mmol; 3 eq)
Yield:		5.37 g (77.0%)	

Gel permeation chromatography

Gel permeation chromatography (GPC) was performed on a Polymer Standard Service PSS (Mainz, Germany) system. Specifications: pump mod. 1260 infinity, MDS RI-detector mod. 1260 infinity (Agilent Technologies, Santa Clara, California, USA), precolumn: 50 x 8 mm PSS PFG linear M; 2 columns: 300 x 8 mm PSS PFG linear M (particle size 7 µm; pore size 0.1 – 1.000 kg/mol) with hexafluoroisopropanol (HFIP, containing 3 g/L potassium trifluoroacetate (KTFA)) as eluent calibrated with PEG standards (molar masses from 0.1 kg/mol to 1000 kg/mol). The columns were held at 40°C and the flow rate was set to 0.7 mL/min. Dried polymer powders were dissolved in eluent and filtered through 0.2 µm PTFE filters (Rotilabo, Karlsruhe, Germany).

Differential Scanning Calorimetry (DSC)

All measurements were performed using aluminum crucibles on a calibrated DS 204 F1 Phoenix system from NETZSCH (Selb, Germany) equipped with a CC200 F1 controller unit from -50°C to 200°C with three heating and two cooling phases and a cooling rate of 10°C/min. The third heating cycle was used to analyze the glass transition temperature of dried polymer powders.

Rheology

All experiments were performed using an Anton Paar (Ostfildern, Germany) Physica MCR 301 system utilizing a plate-plate geometry (25 mm diameter) equipped with a solvent trap and Peltier element for temperature adjustment. All aqueous 15 wt.% samples were dissolved at room temperature stirring constantly and incubated at 5°C for 48 h. In addition, pictures were taken to visualize the gels. A temperature-sweep was performed in oscillation mode from 5-50°C (heating rate: 0.05°C/s) using a fixed amplitude of 0.1% and angular frequency of 10 rad/s. The long-time gelation experiment at 5°C was performed at an amplitude of 0.1% and an angular frequency of 10 rad/s for several hours.

Transmission electron microscopy

For transmission electron microscopy (TEM) experiments, the polymers were dissolved in DI water to a final concentration of 20 g L^{-1} and stored at room temperature. 400 mesh copper–rhodium grids (maxtaform) with a homemade carbon layer were glow discharged in air for 1.5 min at medium power in a Harrick PDC-002 plasma cleaner. The 20 g L^{-1} sample was diluted (1/125 or 1/625) and 8 µL were

incubated on the grids for 1 min before blotting (Whatman filter paper No. 50). The grids were washed with water (three times) and 2% w/v uranyl acetate (three times). After the last dose of uranyl acetate was applied, the grid was left to incubate for 5 minutes before blotting. A single-tilt room temperature holder in an FEI Tecnai G2 Spirit TWIN transmission electron microscope equipped with a tungsten emitter at 120 kV was used. Images were recorded with an Eagle CCD camera under low-dose conditions. The micrographs were binned two times resulting in a pixel size of 2.2 Å per pixel at specimen level.

Small- and wide-angle X-ray scattering (SAXS, WAXS)

SAXS and WAXS experiments were carried out using an in-house setup, which was built by Fraunhofer EZRT (Fürth, Germany). It consists of a MicroMax-007 HF X-ray source (Rigaku, Japan) and a Eiger R 1M detector unit (Dectris, Switzerland). The sample-detector distance can be varied between 5 cm and 3.5 m, which corresponds to possible Q-values between 0.005 and 5 $Å^{-1}$. The complete setup is operated in a vacuum below 0.1 mbar to reduce air scattering. The sample solutions were placed in quartz capillaries (inner diameter: 1 mm, wall thickness: 10 µm) (Hampton Research, Aliso Viejo, California), which were positioned perpendicularly to the X-ray beam. The presented experiments were done at sample-detector distances of 57 mm, 565 mm and 1560 mm with an integration time of 15 min for the shortest distance and 240 min for the two longer configurations. All distances were calibrated using a silver behenate standard sample. For each sample, data was acquired for different temperatures between 5°C – 50°C. To achieve thermal equilibrium, the sample (10 wt.% aqueous solution) was kept at the desired temperature for 15 min prior to each measurement. The SAXS data, which was obtained at the two largest distances, was calibrated in terms of absolute intensities using glassy carbon as a secondary calibration standard.[2-3] The scattering curves of the hydrogels were obtained by azimuthal integration taking the samples thickness, X-ray transmission, detector accuracy, setup geometry and solvent scattering into account following the standard procedures described in the literature.[4]

Micro differential scanning calorimetry (micro-DSC)

Micro differential scanning calorimetry measurements were conducted with a Malvern MicroCal PEAQ-DSC microcalorimeter. The heat of the sample was measured relative to pure water and the enthalpy values were normalized to the molar concentration of the aromatic repeat units. After complete dissolution, the samples were stored in the refrigerator at 4°C for about 48 h, degassed at

5°C, transferred to the instrument precooled at 2°C or 10°C, and kept at the temperature for different times, as indicated, prior to heating. Each sample was heated with the rate of 1°C/min to 100°C, after which they were cooled again to the starting temperature with the same rate.

Fluorescence spectroscopy

2-(4-Dimethylamino)styryl)-1-methylpyridinium iodide (DASPMI) was purchased from Molecular Probes Inc., Life Technologies and used without further purification. 4,4´-Difluoro-4-bora-3a,4a-diaza-*s*-indacene meso-substituted with para-dodecylphenyl moiety (BPC12) was synthesized in our group according to literature methods.[5] Gibco™ Dulbecco's phosphate-buffered saline (DPBS) pH 7.25 was purchased from Thermo Fisher Scientific (Massachusetts, USA).

Steady-state fluorescence. The fluorescence spectra were obtained using a FLS-1000 spectrofluorometer (Edinburgh Instruments, UK) equipped with a temperature controlled cuvette holder. The excitation wavelengths were 490 nm for BPC12, 460 nm for DASPMI.

Time-resolved fluorescence. Fluorescence intensity decay curves were measured using time-correlated single photon counting (TCSPC) system (PicoQuant, GmBH) as described earlier.[6] The samples were excited at 483 nm with LDH-P-C-485 laser head. Excitation slight scattering was reduced by use of cutoff filter > 490 nm. Monitoring wavelengths for the decays were 520 nm for BPC12 and 580 nm for DASPMI. The measurements were performed in quartz cuvettes with a 1 cm path length, measured in a cuvette holder equipped with temperature controlling module. The fluorescence decays were deconvoluted with the instrumental response to give the fluorescence lifetime data with the time resolution of ~ 100 ps (Figure S3.1, left).[6]

Viscosities determination. Viscosities were calculated at 5, 12, 22 and 37°C by the eq. S1

$$\log \tau_f = \log \frac{z}{k_r} + \alpha \log \eta \qquad \text{(S1)}$$

where τ_f – fluorescence lifetime of BPC12/DASPMI in the solution of a given viscosity η , and z and α are constants. The linear part of the *log* τ_f versus *log* η dependencies is taken as calibration plot and the viscosities are calculated using the equations of the linear fits. Calibration plots and equations for DASPMI are shown in (Figure S3.1, right) and those of BPC12 rotor are taken from the literature (Table S3.1).[7]

Four series of nine water/glycerol mixtures of different viscosities at concentrations of glycerol between 80 wt.% and 100 wt.% were prepared as a calibration set. Viscosities (η) of each binary

mixture were measured at 5, 12, 22 and at 37°C using a LOVIS 2000M rolling ball microviscosimeter from Anton Paar (Graz, Austria) with a LOVIS 1.8 capillary and a steel ball of 1.5 mm diameter. Prior to viscosity measurements, the density of the sample at the specific temperature was recorded using a DMA 4100M density meter from Anton Paar (Graz, Austria). Each water/glycerol mixture was used to dissolve 5 µM of DASPMI and the corresponding fluorescence intensity decays were recorded in quartz cuvettes using the TCSPC system (PicoQuant, GmBH) described above (Figure S3.1, left). Linear calibration plots of the solvent viscosity versus fluorescence lifetime were obtained at each temperature in double logarithmic scale (Figure S3.1, right).

Raman spectroscopy

The Raman spectra were recorded on an alpha 300R$^+$ confocal Raman microscope from WITec GmbH (Ulm, Germany) equipped with a 50x objective (NA 0.8, Epiplan Neofluar, Zeiss, Germany) and a 532 nm laser (39.4 mW). A 20 wt.% sample was measured after equilibration at 5°C and 40°C using a temperature controllable Peltier stage (LTS 120, Linkam Scientific Instruments Ltd., Tadworth, UK). Spectra are shown as an average spectrum of 3 spectra at different locations on the same sample, which were recorded with an integration time of 5 s and 10 accumulations. The resulting data were processed with cosmic ray removal and background subtraction. The bulk water signals at 3100 cm^{-1} - 3700 cm^{-1} were fitted using a Gaussian deconvolution method as described elsewhere.[8-9]

Nuclear magnetic resonance (NMR) experiments in solution

All experiments in solution were performed at a Bruker Avance III HD 600 spectrometer (Karlsruhe, Germany) operating at 600.4 MHz equipped with a BBFO 5 mm probe using a BCU-02 temperature control unit. ^{1}H NMR experiments of a 20 wt.% A-pPheOzi$_{15}$-A sample in D_2O were acquired with a 30 ° flip angle and 8 or 16 scans without sample spinning. A series of variable temperature experiments was performed in the range from 2°C to 39.15°C in a step size of 3 to 5°C. The sample was kept at the desired temperature for 10 minutes prior to each measurement. Temperature calibration was done using 4 % MeOH in MeOD and 80 % ethylene glycol in DMSO-d$_6$. All recorded spectra were referenced using the temperature dependent HDO signal.

2D ^{1}H-^{1}H nuclear Overhauser effect NMR spectroscopy (NOESY) experiments at 5°C and 40°C were recorded using the noesygpphpp pulse sequence (scans: 32, t_1 increments: 256, relaxation delay: 2.5 s). To ensure discrimination between cross-relaxation and spin-diffusion different mixing times (40 µs,

60 µs, 80 µs, 150 µs and 250 µs) were used. For better visualization of the aromatic region, phase and baseline correction of 2D data was confined to the range of 6-8 ppm and TDeff was set to 2867 in the f2 dimension. Additionally, 1D slices of relevant aromatic regions were extracted.

Low field nuclear magnetic resonance

Low field NMR measurements were performed using a Bruker Minispec mq20 (Bruker, Karlsruhe, Germany) operating at 19.9 MHz with a 90° pulse length of 2.5 µs and a dead time of about 15 µs. 20 wt.% polymer samples in D_2O were prepared and transferred to 10 mm sample tubes reaching a maximum filling height of 8 mm to restrict the sample to the region of homogenous B_0 field. Spin-Spin (T_2) relaxation times were measured by detecting a basic FID after a 90° pulse covering the initial short-time decay (first ca. 200us), using a magic sandwich echo pulse sequence to overcome the receiver dead time and a time-incremented Hahn-Echo pulse sequence for slowly relaxing components. Multiple-quantum (MQ) NMR using a pulse sequence based on the experiment of Baum and Pines was applied to probe the overall magnitude of residual dipolar couplings in chains subject to restricted conformational dynamics.[10]

Solid-state nuclear magnetic resonance

Solid-state NMR (ssNMR) measurements were performed using a 4 mm double-channel Bruker probe at 9.4 T using between 3 and 7 kHz magic angle spinning (MAS). The hydrogel sample was cooled to 273 K prior to measurement. For the ^{13}C CP MAS experiment, a 2 ms ramp (50 to 100 %) on the ^{1}H channel was used during the cross-polarization (CP) contact time for all samples. ^{13}C NMR spectra with direct excitation were recorded with short interscan delays of 1 s to probe mobile components. For heteronuclear decoupling during acquisition, SPINAL64 was employed with a 100 kHz nutation frequency (^{1}H). The chemical shifts were referenced using adamantane (left signal at 38.48 ppm) by subsequent adjustment of the magnetic field.

Molecular modeling

Three systems, each containing eight chains of a single polymer type, were modeled: Me-pMeOx$_{35}$-*b*-pPheOzi$_{15}$-*b*-pMeOx$_{35}$-EIP, Me-pEtOx$_{35}$-*b*-pPheOzi$_{15}$-*b*-pEtOx$_{35}$-EIP or Me-pMeOzi$_{35}$-*b*-pPheOzi$_{15}$-*b*-pMeOzi$_{35}$-PipBoc (Me = methyl group, EIP = ethyl isonipecotate, PipBoc = 1-Boc-piperazine). The

hydrophobic pPheOzi blocks faced each other to form a single inner strand along the Z axis and the hydrophilic A-blocks were bent outwards. Four individual molecules made up the strand and were subsequently duplicated and moved next to the original polymers along the Z axis, ultimately resulting in two layers of polymers in each simulation box. The stretched-out, hydrophilic A-blocks were subjected to an energy minimization and a short, 50 ps long simulation with the Noisé-Poincaré-Andersen method[11-12] (applying the Amber14:EHT force field[13-14] with the R-field implicit solvation model[15]) to yield a more compact starting conformation, while keeping the inner strand in a straight orientation (Figure S8.1). All modeling was performed with MOE (Molecular Operating Environment 2019.01).[16] This setup was inspired by previous modeling studies regarding worm-like micelles of small molecules, in which the generation of a continuous micelle was also achieved *via* initial placement of hydrophobic parts in the inner and hydrophilic moieties in the outer regions of the threadlike structure, which was aligned along one axis of the simulation box.[17-19] RESP partial charges[20] of single monomers used as building blocks were derived from calculations with Gaussian 09 Rev. C.01[21] (Hartree-Fock level of theory, 6-31G* basis set); parameters based on the Amber14ffSB[13] and GAFF2[22] force fields were assigned *via* antechamber and parmchk2 of AmberTools18.[23-24] During charge derivation monomers were capped with residues of the same type and terminal groups with an A-block monomer. The calculated parameters were used to generate polymers with an initial straight conformation using tleap.[23-24]

Starting structures were solvated with TIP3P water[25] in a simulation box with a minimum border-to-polymer distance of 20 Å in the X and Y directions. Water molecules found inside the inner hydrophobic strand after this initial placement were removed if the distance to the pPheOzi blocks was less than 10 Å. Periodic boundary conditions with minimum image convention were applied during simulations, which allowed for infinitely sized worm-like micelles along the Z axis and ensured a sufficient distance between polymers of neighboring boxes along the X and Y dimensions. Simulations were performed using NAMD 2.13[26] with 2 fs time steps. An initial energy minimization of 10,000 steps was conducted before slowly heating the system from 100K to 278K over the course of 500 ps. Harmonic constraints were initially applied on all polymers and gradually reduced over an additional 1.6 ns, allowing rapid reordering of solvent molecules around the polymers. Langevin dynamics and the Nosé-Hoover Langevin piston method (1 atm) were used for temperature and pressure control in an NPT ensemble. After another 2 ns of additional equilibration, the production run was performed for 600 ns. Semi-isotropic coupling allowed for fluctuations along the Z axis, independent from the X and Y axes. The particle mesh Ewald method[27] with a cut-off of 1.2 nm was applied and coordinates were saved every 10 ps. Subsequent analyses were performed using CPPTRAJ.[28] Average densities for polymer groups

around pPheOzi monomers were retrieved as follows: All pPheOzi residues were iteratively aligned onto the same monomer. Next, binned occupancy histograms of different moieties around the center of the aligned residue were calculated for the last 100 ns using the grid command in CPPTRAJ. This was performed on a 1.6 x 1.6 x 1.6 nm^3 grid with a 1 Å resolution. After this procedure, the obtained values around each pPheOzi monomer were added up at each grid element and divided by the number of analyzed frames (10000) and monomers (104). Thus, densities represent the average amount of atoms of interest found at each grid element per frame around a single monomer. The first and last pPheOzi monomer of each pPheOzi block (16 out of 120) were excluded from this calculation, as these are always situated near neighboring A-blocks. Additionally, several distances between these pPheOzi monomers and the other polymer residues were analyzed, as well as the angle ω between the plane of nearby amide (N-(C=O)-C) groups and the phenyl ring plane for every 10 ps of the last 100 ns.

Complementary Material

Chapter S1 – SAXS and WAXS measurements

The intensity I as a function of Q from the SAXS measurements was plotted for different temperatures for **P1**. In the hydrogel state (5°C, blue), a pronounced structure peak (Figure S2.1 A) is followed by two clearly defined regions with different slopes (arrowheads). These regions at intermediate and high Q-values can be assigned to different self-assembled species. Using a power-law expression the different slopes can be determined, indicating the presence of spherical micelles and worm-like micelles in the gel state.[29] Above T_{gel}, the second species at high Q-values (worm-like micelles) disappears, confirming that the order-order transition found at low concentration also occurs at higher concentration. In addition, the structure peak is shifted towards higher Q-values indicating a lower particle/particle distance in the sol state due to the formation of small spherical micelles (vertical lines). This is a logical consequence of the disintegration of relatively few worm-micelles into much more numerous spherical micelles. The extrapolation of the absolute intensity I to Q_0 (horizontal lines) was used as a measure for relative mean particle size. In the gel state (5°C), a higher $I(Q_0)$ value compared to the liquid state was observed, indicating once more larger particles in gel state (worm-like micelles) in comparison to the sol state (spherical micelles). The qualitative analysis of the SAXS scattering profiles is summarized in more detail in Table S1 below.

Table S1. Summary of SAXS analysis for a 10 wt.% **P1** hydrogel sample at different temperatures.

Temperature [°C]	Intensity of Q→0[a]	2π/Q(I_{max})[b] [nm]	Slope at intermediate Q-value	Slope at high Q-value
**5**	0.272±9.5x10^{-5}	22.3	-5.1±1.2x10^{-3}	-2.2±2.1x10^{-4}
**25**	0.318±1.02x10^{-4}	23.9	-4.9±1.3x10^{-3}	-2.3±2.4x10^{-4}
**32**	0.210±8.5x10^{-5}	20.6	-4.9±4.6x10^{-4}	/
**40**	0.184±8.0x10^{-5}	19.8	-5.1±4.5x10^{-4}	/
**50**	0.187±8.1x10^{-5}	20.5	-5.2±4.5x10^{-4}	/

[a]Extrapolation of intensity to Q_0; [b]*Q*-value at maximum *I* value (structure peak)

Additionally, temperature-dependent wide angle X-ray scattering of **P1** (Figure S2.1 B) can provide insights into intra- und interpolymer interactions as previously described for biopolymers[30] and thermogelling peptide.[31] In the gel state (5°C, blue), a rather defined peak centered around 4.2 Å was observed, which could be interpreted to hint towards π-π interactions of the phenyl moieties.[32] With increasing temperature, the peak position was maintained, but a noticeably broadening between 4.2 and 7 Å indicates reduced order and increased degrees of freedom.

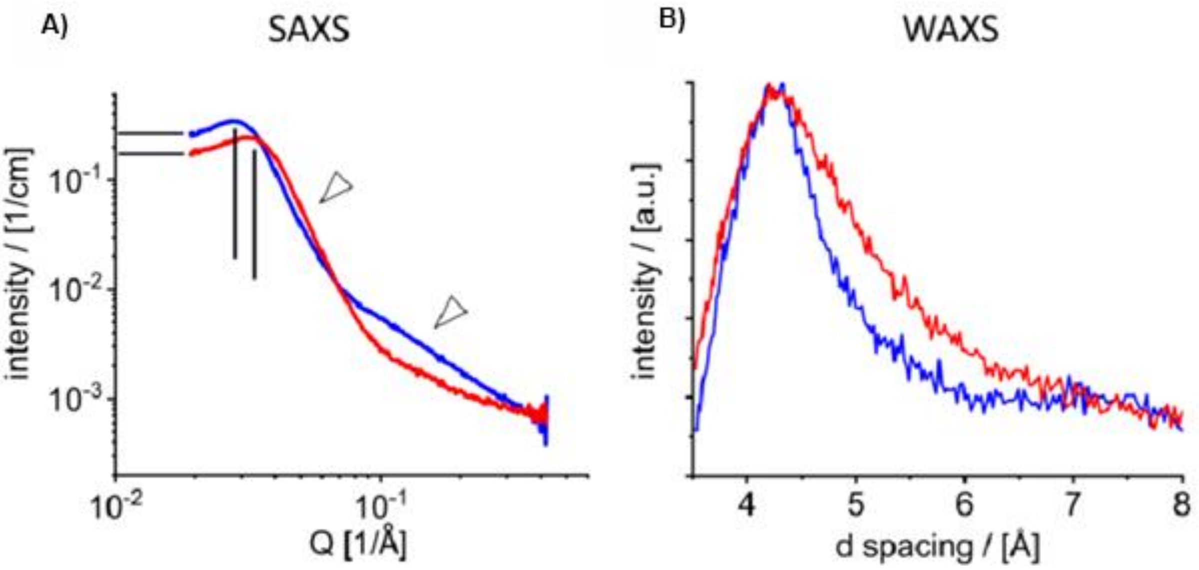

Figure S1.1. A) SAXS scattering curves of aqueous solutions (10 wt.%) of **P1** at 5°C (blue, hydrogel) and 40°C (red, liquid). Two defined slopes regions can be defined (triangles). Further, the position of the structure (vertical lines) and absolute intensity of Q_0 →0 (horizontal lines) are different at 5°C and 40°C. B) WAXS analysis of aqueous solutions (10 wt.%) of **P1** at different temperatures (blue: 5°C, red: 40°C) in the d spacing region of 3.4-8 Å at 100 g/L.

Chapter S2 – Polymer characteristics

Table S2.1 Characteristics of the polymers pMeOx-*b*-pPheOzi-*b*-pMeOx (**P1**), pEtOx-*b*-pPheOzi-*b*-pEtOx (**P2**) and pMeOzi-*b*-pPheOzi-*b*-pMeOzi (**P3**).

Polymer	M_n [kg/mol][a]	Đ[b]	Hydrophobic/hydrophilic ratio[c]	T_g [°C][d]
**P1**	8.5	1.19	4.4	76.9
**P2**	9.5	1.21	4.4	62.5
**P3**	9.6	1.49	4.7	41.5

[a]Theoretical M_n value. [b]Dispersity obtained *via* GPC experiments. [c]Obtained *via* integration of specific signals in ^{1}H NMR experiments. [d]Glass transition temperature obtained *via* DSC experiments.

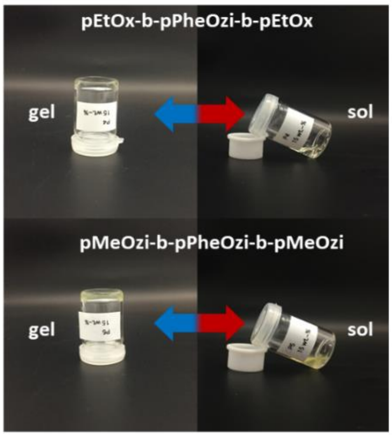

Figure S2.1 Pictures of aqueous samples (15 wt.%) of **P2** and **P3** in gel (5°C) and sol (40°C) state. Gelation was carried out for 24h at the fridge.

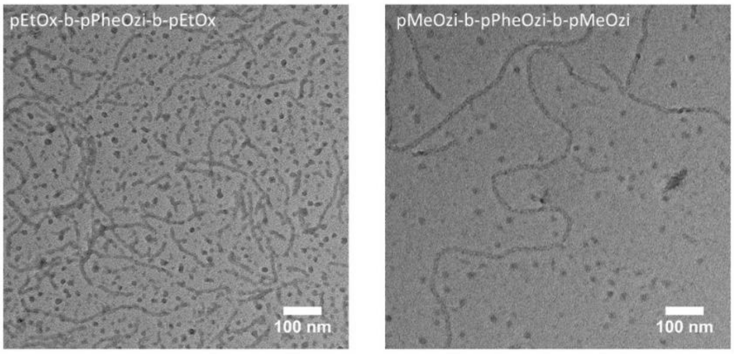

Figure S2.2 TEM images of aqueous solutions (5 wt.%) of **P2** and **P3**. Polymer **P3** only showed "isolated" worms with no pronounced crosslinking between individual worm-like micelles.

Chapter S3 – Fluorescence spectroscopy of molecular rotors

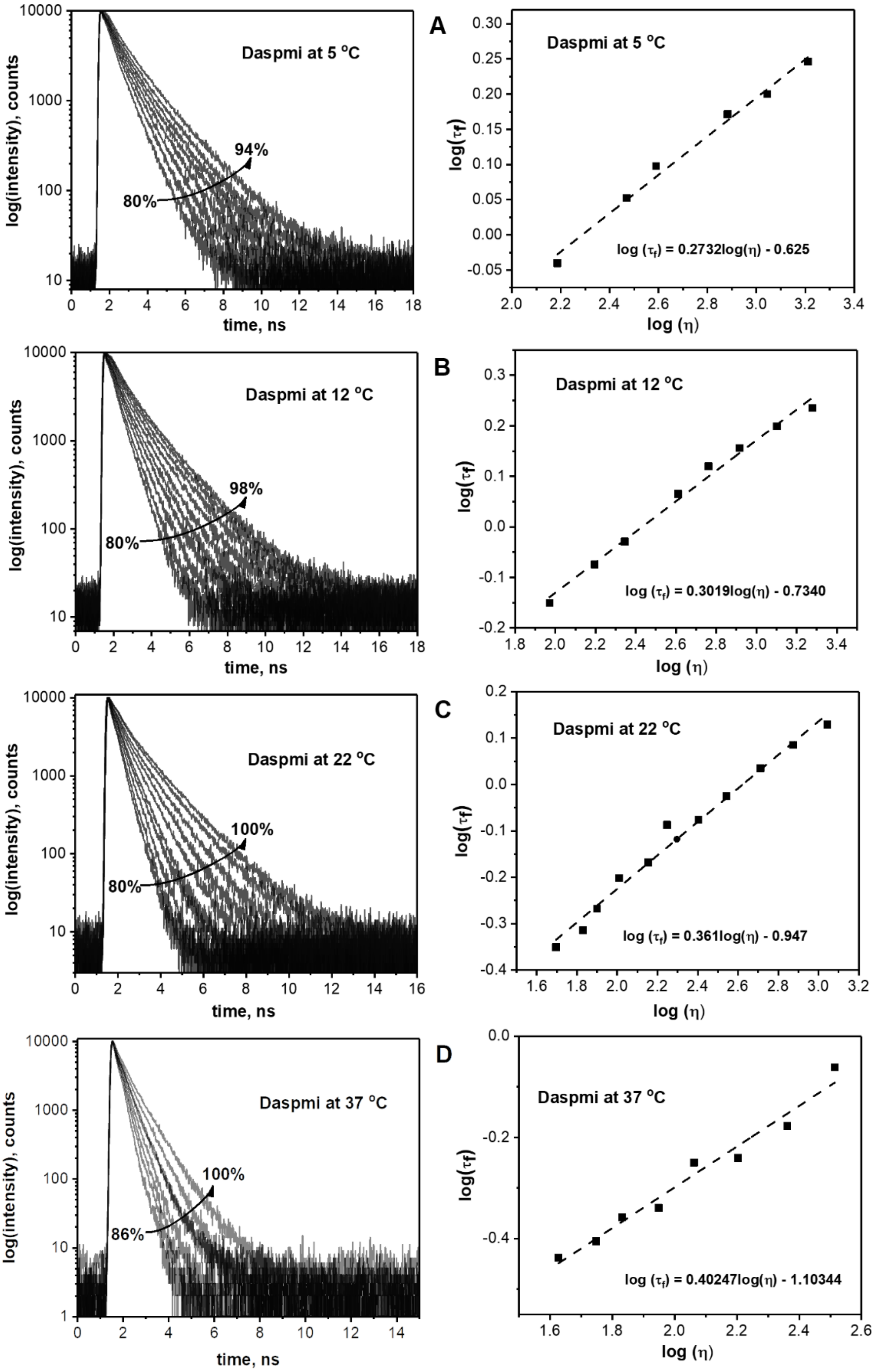

Figure S3.1: Kinetic curves (left) and corresponding calibration curves (right) of DASPMI (5 µM) in series water/glycerol mixtures of different viscosities at 5 (A), 12 (B), 22 (C) and 37°C (D).

Table S3.1: Calibration equations for BPC12 in methanol-glycerol mixtures of different viscosities:[7]

Temperature	Calibration equation
5°C	log (t) = 0.5571 log (h) - 0.9444
12°C	log (t) = 0.6072 log (h) - 1.1338
22°C	log (t) = 0.6073 log (h) - 1.0987
37°C	log (t) = 0.6696 log (h) - 1.1898

Fluorescence intensity together with the shift of fluorescence spectrum maximum provides information about the phase transition of the polymer as well as the polarity changes of the probe microenvironment. For example, changes in the fluorescence intensity and wavelength of DASPMI have been attributed to sol-to-gel transition as well polarity changes of the microenvironment.[6, 33] Polarity-sensitive properties of BPC12 and its derivatives have also been noticed, although with a lower degree of responsiveness.[34] The same probes have been used to determine microviscosities of hydrophylic (DASPMI) and hydrophobic (BPC12) compartments of the self-assembled systems.[6] With molecular rotors such as these, the viscosity sensitivity can be seen as changes in the fluorescence lifetime, which is affected by the rotation ability of the structural segments with respect to each other. This in turn is strongly dependent on the immediate molecular environment. However, it has to be kept in mind that it is not always clear what this immediate molecular environment is exactly.

For both DASPMI and BPC12 series in **P1** and **P2** shorter living component of the biexponential fitting is presented (Figure S3.2) and was used for further viscosity calculation as only the component showed viscosity dependent behavior upon temperature change. The shorter living component was attributed to free BPC12 or DASPMI, which reflects the dye mobility and depends on the dye environment (viscosity). Longer living component stayed almost constant for both dyes (~3 ns for DASPMI and ~7 ns for BPC12) regardless the temperature and was attributed to a more hindered molecular rotation corresponding to an interaction (probably complex formation) of the dyes with polymer molecules. Steady-state and time-resolved fluorescence measurements revealed that 20 wt.% **P3** solution had an intrinsic fluorescence upon 483 nm laser excitation, and fluorescence overlapped with that of both used dyes. Two lifetimes were resolved at both DASPMI (580 nm) and BPC12 (520 nm) detection wavelengths in background measurements of the pure polymer (Figure S3.2, gray trends). These lifetimes were fixed in multiexponential fitting for the polymer samples with dissolved dyes. For DASPMI-containing sample only one extra lifetime was resolved (Figure S3.2, dashed red trend), while in the presence of BPC12 - two extra lifetimes were calculated (Figure S3.2, blue trends). Similar to the situation observed for **P1** and **P2**, BPC12 long-living (~ 5.5 ns) component was assigned to a non-

covalent complex formation of the dye and the polymer molecules, and the shorter living component was used for further viscosity calculations (Figure S3.2, dashed blue trend).

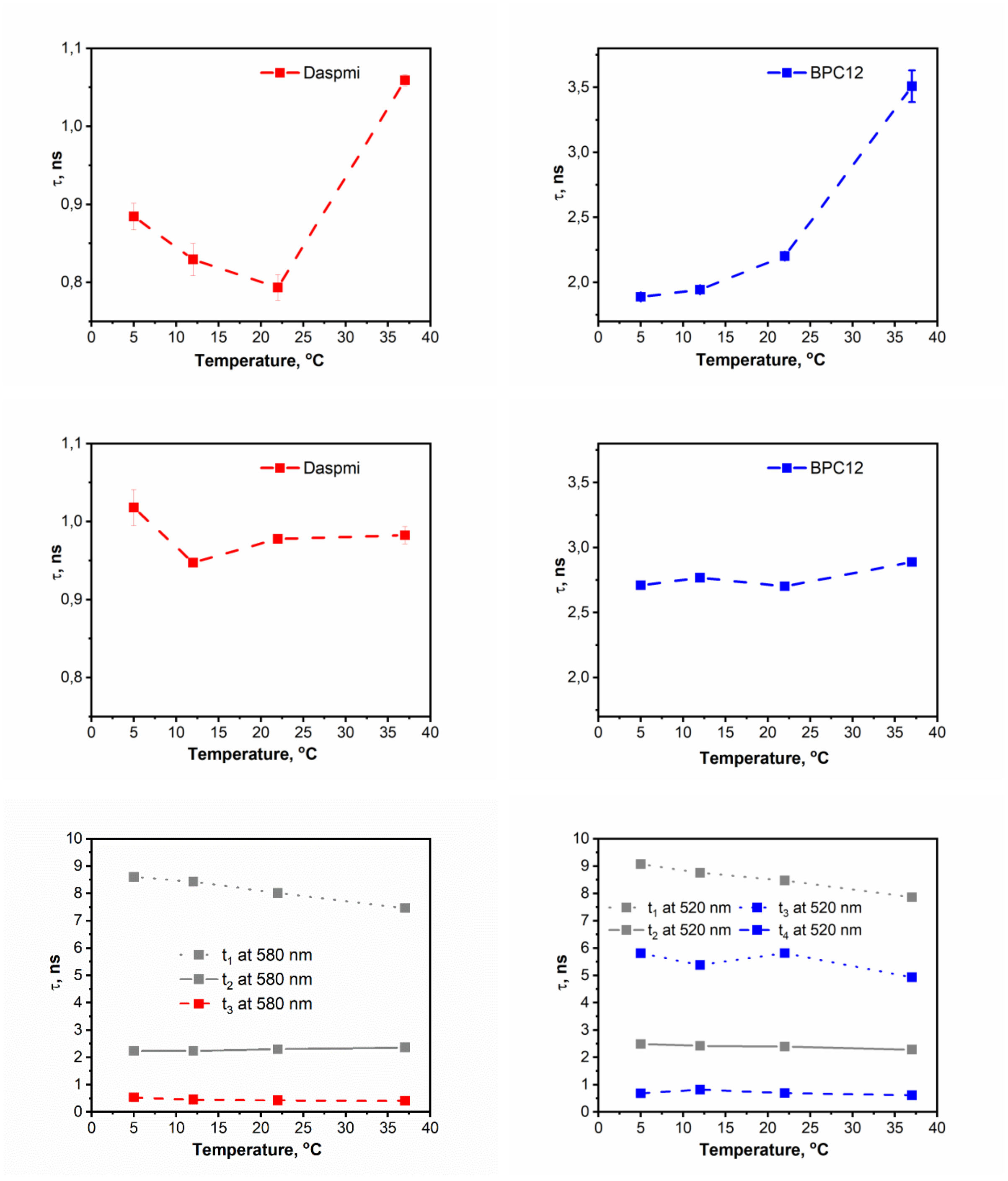

Figure S3.2: Fluorescence lifetimes of 5 µM DASPMI (left) and 5 µM BPC12 (right) in 20 wt.% **P1** (top row) and **P2** (middle row) block copolymer solutions at different temperatures calculated by biexponential fitting. Fluorescence lifetimes of 5 µM DASPMI (left) and 5 µM BPC12 (right) in 20wt.% **P3** (low row) solution at different temperatures calculated by tri- and four-exponential fitting, respectively. Gray trends are lifetimes of pure **P3**, red and blue trends are lifetimes correspondingly attributed to DASPMI and BPC12.

Microviscosities in the sol and gel states were determined by fluorescence lifetime data after appropriate calibration (Figure S3.1, Table S3.1) at four temperatures (5, 12, 22 and 37 °C). In contrast to bulk viscosity (macroviscosity) of **P1**, which is higher in the gel state, higher microviscosities were obtained in the sol state and the hydrophilic probe gave almost two times higher values than the hydrophobic one. The microviscosities decreased upon gelation and were similar for both probes below 25 °C (Figure S3.3). In case of **P2**, a similar but less pronounced trend was observed for DASPMI *i.e.* a decrease in microviscosity upon gelation, while for BPC12 the microviscosity remained almost the same throughout the whole temperature range. This observation may indicate that upon gelation of this copolymer, the condensation takes place in the hydrophilic corona, but the hydrophobic core is less affected during spheres-to-worms transition. Moreover, it seems that slightly lower temperatures are needed for gelation of **P2** compared to **P1** (Figure S3.3. dashed lines) that is in agreement with macroscopic viscosity results for the polymers. Microviscosities calculated for both hydrophilic and hydrophobic compartments of **P3** upon thermogelation are the lowest measured in this study. In striking contrast to previous block copolymers, no significant changes in the microviscosity of neither DASPMI nor BPC12 upon order-to-order transition of this copolymer could be noticed. This suggests, albeit indirectly, a crucial role of hydrophilic block for sphere-to-worm transition and more profound change in hydrophilic compartment of **P1** and **P2** upon thermogelation.

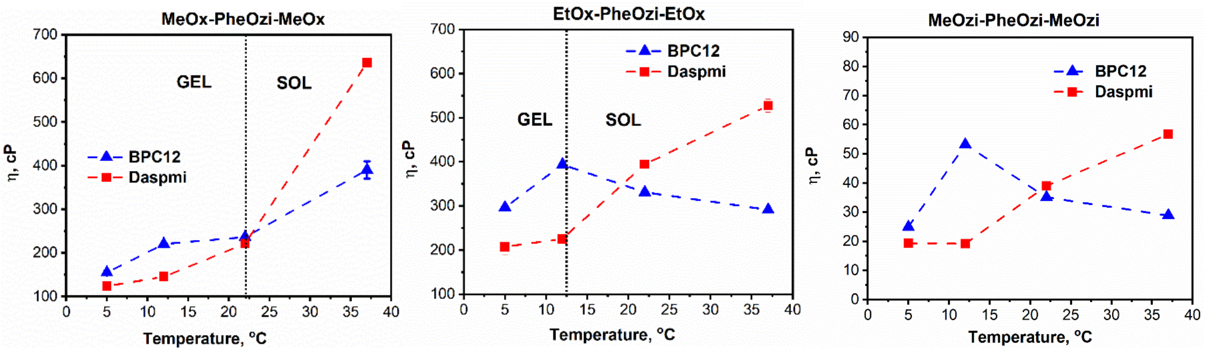

Figure S3.3: Microviscosity values for the microenvironment of DASPMI and BPC12 molecular rotors in a 20 wt.% aqueous polymer samples obtained from fluorescence lifetime experiments as a function of temperature.

Steady-state fluorescence spectroscopy complements the picture. The shift of fluorescence spectrum maxima (λ_{max}) can provide information on polarity changes of the probe´s microenvironment. For both probes, a bathochromic shift was observed at low temperature, indicating an increase in the polarity of the probe microenvironment upon gelation of **P1** (Figure S3.4).[33-34]

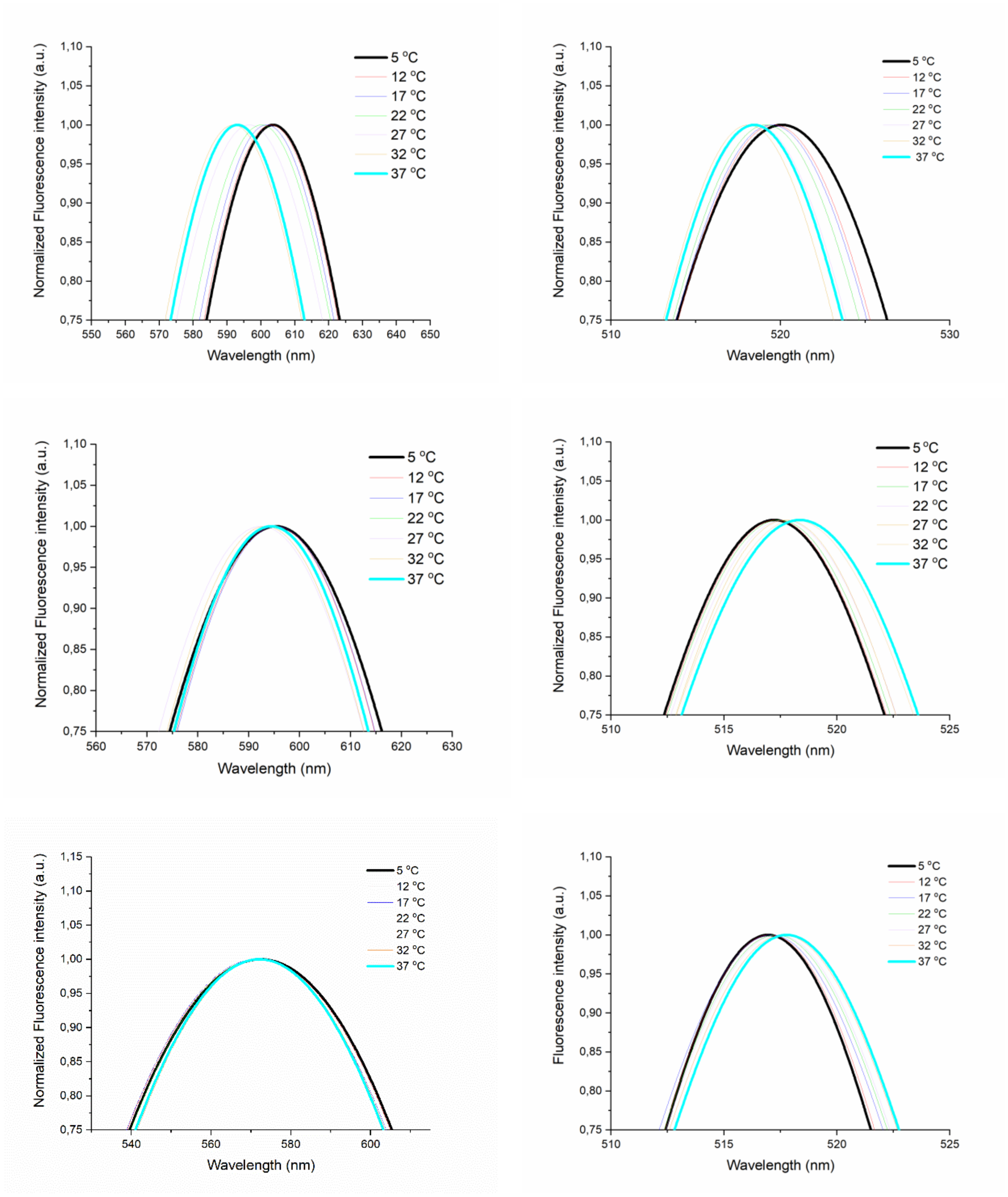

Figure S3.4: Normalized fluorescence spectra of 5 µM DASPMI (left) and 5 µM BPC12 (right) in 20 wt.% **P1** (top row), **P2** (middle), and **P3** solutions at different temperatures.

This increase suggests that the probes can be partially expelled from polymeric self-assemblies and become more exposed to polar aqueous solvent. For **P2**, the change in polarity of the microenvironment for DASPMI is less pronounced while BPC12 does not show significant spectral shift. The bathochromic shift of fluorescence spectrum maximum for DASPMI still indicates that the environment of the probe becomes more polar upon micelles-to-worms transition. An even less

pronounced temperature dependence was observed for both BPC12 and DASPMI when studying **P3** thermogelation (Figure S3.4), where both dyes exhibited negligible hypsochromic shifts. It is noteworthy that the initial polarity of the DASPMI microenvironment in **P3** micelles is significantly lower than that for the other two studied block-copolymers. The conclusion is drawn given the fluorescence maximum of DASPMI in **P3** micelles is centred at ca. 570 nm, which is 20 nm shorter than those of **P1** and **P2** sols.

Ultimately, the time-resolved and steady-state fluorescence measurements clearly show that the microenvironment of both molecular rotors is more polar and less viscous in the gel state, suggesting that the gelation of **P1** causes a probe migration out of the condensed polymeric assembly closer to the polymer-water interface. In the case of **P2** this effect can be seen only for DASPMI, which is presumably co-localized in the hydrophilic shell, indicating the crucial role of EtOx moieties upon thermogelation. It seems that rather weak intermicellar interactions of **P3** do not lead to any changes in the microenvironment of either dye in terms of polarity or microviscosity.

Chapter S4 – Raman measurements

In the gel state of **P1**, a sharp and moderately intense signal is observed at 731 cm^{-1}, which is much weaker and barely resolved in the sol state. For **P2**, also a moderate signal at 731 cm^{-1} is observed that is slightly weaker in the sol state, while a signal at 739 cm^{-1} barely distinguishable in the gel state becomes markedly stronger in the sol state. For **P3**, we observed a clear peak at 804 cm^{-1} in the gel state, which is absent in the sol state. In this spectral region, we expect C-C stretching modes, which are abundant in our polymers. At this point, we cannot hypothesize on the assignment of this signal without further understanding of the system. All three polymers show strong signals around 1000 cm^{-1}, attributed to the phenyl ring and the C-H in plane bending mode. Interestingly, for **P1** and **P2**, the main signal shows a hypsochromic shift, while in **P3**, we observe a bathochromic shift. At 1464 cm^{-1}, a small but clearly distinguishable peak is exclusively present in the gel state of all polymers. Unfortunately, both aromatic ring vibrations as well as CH_3 and CH_2 deformation vibrations ubiquitous in the polymer backbone and hydrophilic sidechains appear in this region, making an unambiguous assignment challenging.

In addition, for **P1** and **P2**, a clear difference between sol and gel state is also observed in the OH region of 3100 cm^{-1} to 3600 cm^{-1}, originating from water molecules. The different types of bonding modes in water molecules can be categorized using Gaussian deconvolution to divide the OH region into areas with different binding strength.[8, 35] For **P1**, this suggests that water is less mobile in the hydrogel

compared to the sol (Figure S4.1B) as indicated by the increased contribution of the peak at 3250 cm^{-1} (Figure S4.1B, red line, 1). Qualitatively, the situation for **P2** appears very comparable. For **P3**, the water evaporated too quickly at 40°C and we could not obtain suitable spectra despite various adjustments in measurement parameters.

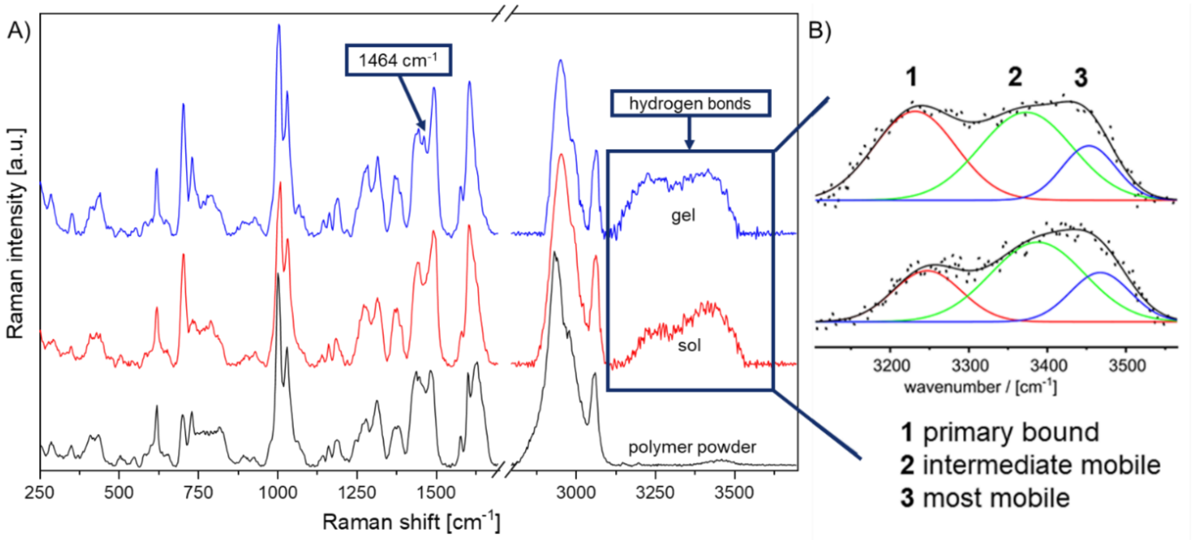

Figure S4.1 Temperature dependent Raman experiments of a 20 wt.% sample of **P1** in sol (40°C) and gel (5°C) state. A)) Normalized Raman spectra (250-3700 cm^{-1}) of dried **P1** powder (black), **P1** sol (40°C, red) and **P1** gel (5°C, blue). At 1464 cm^{-1} a peak appears in the gel state, with additional changes in the signal pattern in the region of hydrogen bonds involving water molecules (3100-3600 cm^{-1}). B) Deconvolution of the OH-region of the spectrum (dashed line) using the Gaussian deconvolution method showing the individual Gaussian peaks (colored lines) and their sum (black line). The different possible hydrogen bonds are describing different types of water, which can be assigned to primary bound, intermediate mobile, and most mobile water molecules. Raman shifts ranging from 3200 to 3300 cm^{-1} indicate more strongly H-bonded water molecules (primary bound water), intermediate mobile water found between 3300 and 3400 cm^{-1} and highly mobile water molecules are found between 3450-3550 cm^{-1}.

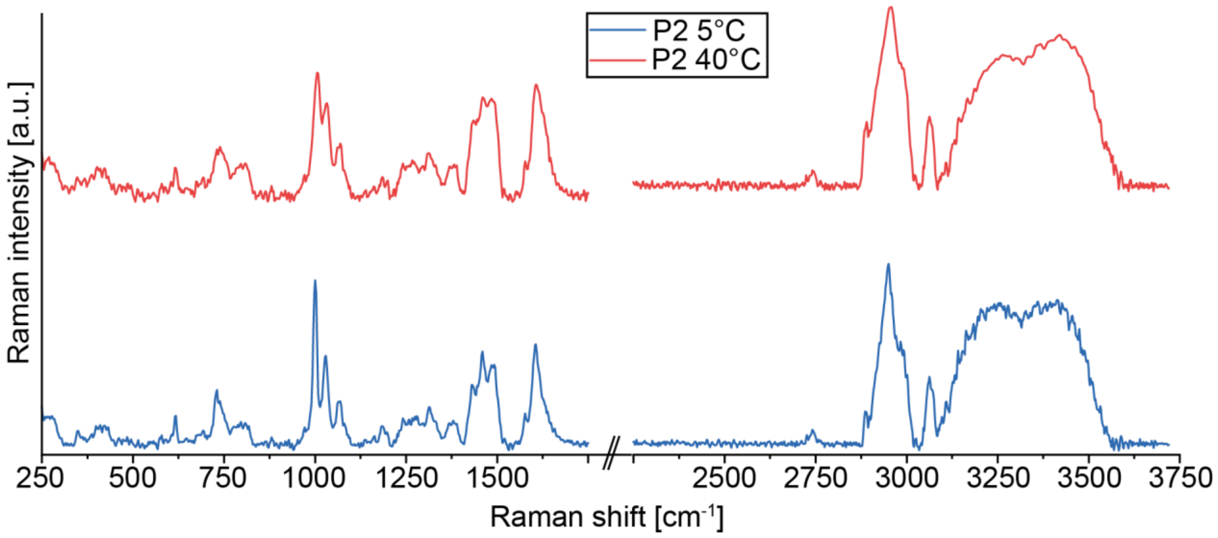

Figure S4.2 Temperature dependent Raman experiments of a 20 wt.% sample of P2 in sol (40°C, red) and gel (5°C, blue) state.

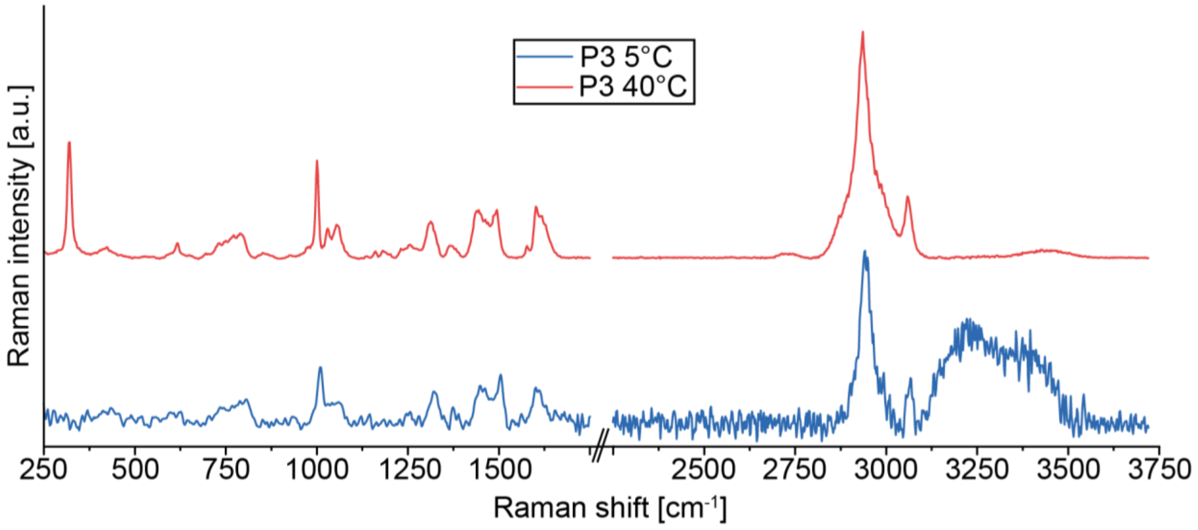

Figure S4.3 Temperature dependent Raman experiments of a 20 wt.% sample of P3 in sol (40°C, red) and gel (5°C, blue) state.

Chapter S5 – NMR experiments in solution

For a more quantitative assessment of the changing ^{1}H NMR signal intensities during the temperature induced phase transition the fraction p was calculated with the integrals $I(T)$ and $I(T_0)$ at the respective temperatures T and T_0 using the following equation (S1). ^{1}H NMR spectra were measured from 5°C to 40°C with the highest signal intensity at 40°C, which is therefore defined as T_0 giving a p-ratio of 0. Decreasing signal intensities at lower temperatures could be quantified by $0 \leq p \leq 1$.

$$p = 1 - \frac{I(T)}{I(T_0)\frac{T_0}{T}} \tag{S2}$$

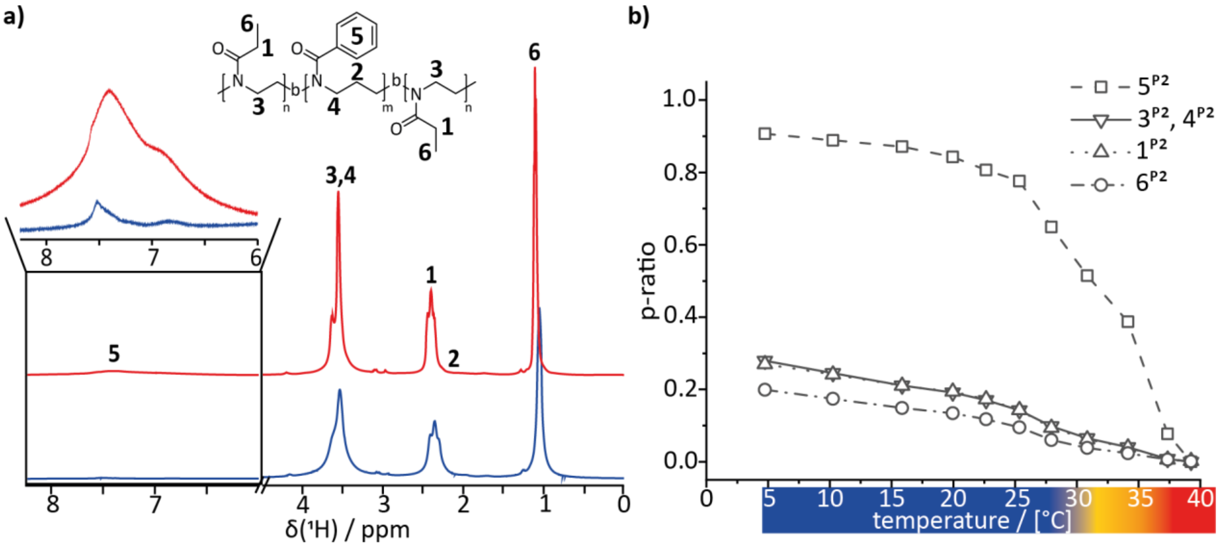

Figure S5.1 a) Structure of the amphiphilic triblock copolymer pEtOx-b-pPheOzi-b-pEtOx (**P2**) including numbering scheme and ^{1}H NMR spectra at 5°C (blue) and 40°C (red) of a 20 wt.% **P2** sample. b) p-ratios for the different proton peak integrals of **P2** as a function of temperature.

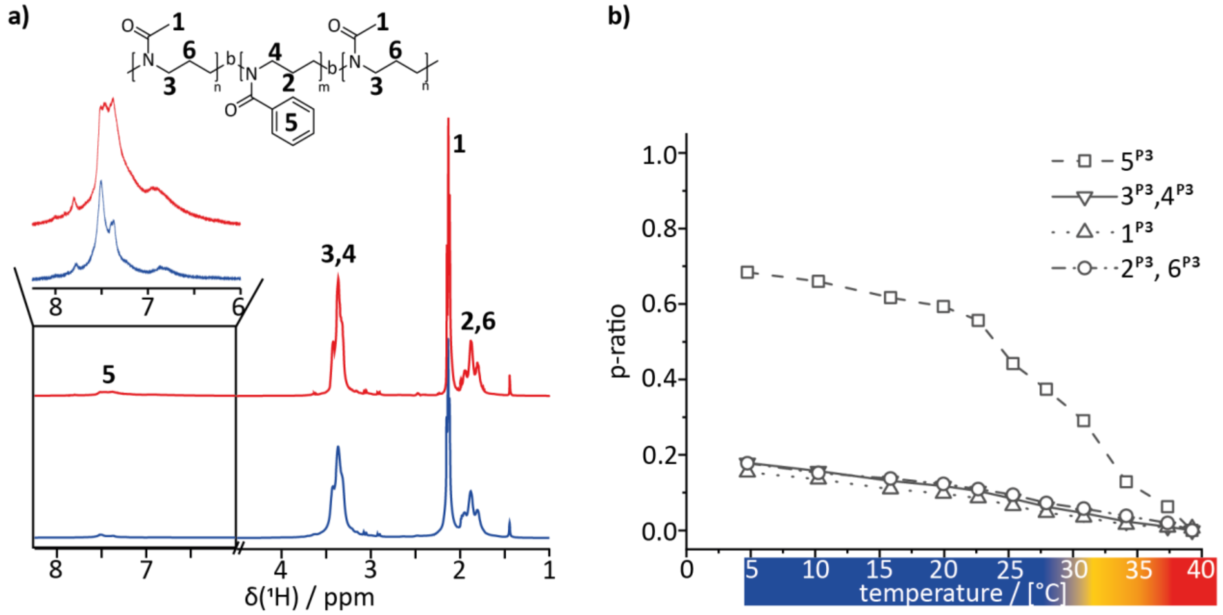

Figure S5.2 a) Structure of the amphiphilic triblock copolymer pMeOzi-b-pPheOzi-b-pMeOzi (**P3**) including numbering scheme and ^{1}H NMR spectra at 5°C (blue) and 40°C (red) of a 20 wt.% **P3** sample. b) p-ratios for the different proton peak integrals of **P3** as a function of temperature.

Spatial proximity between the different chemical moieties resulting in Nuclear Overhauser Enhancements (NOEs) was measured with ^{1}H-^{1}H NOESY NMR in solution for **P1**, **P2** and **P3** at 5°C and 40°C. As no significant changes were observed between the three polymers, spectra of **P1** are shown exemplarily in its gel (left side) and sol state (right side).

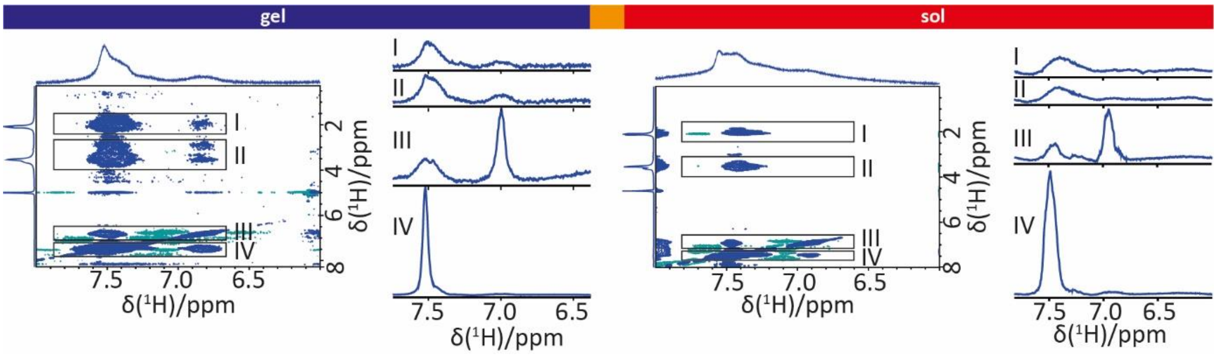

Figure S5.3 Extracts from ^{1}H-^{1}H NOESY NMR experiments of a 20 wt.% sample of **P1** in D_2O recorded with a mixing time of 40 µs, 32 transients, and 256 t_1 increments using a relaxation delay of 2.5 s at 5°C (blue, gel state, worm like micelle) and 40°C (red, sol state, spherical micelle). For signal areas of interest 1D slices are shown on the right of the respective NOESY spectrum. NOESYs of **P1** are shown exemplarily, as similar spectra were obtained for Polymer **P2** and **P3**.

Chapter S6 – Low-field NMR experiments at 20 MHz

The transverse spin-spin relaxation of the 20 wt.% samples of **P1**, **P2** and **P3** in D_2O were measured using a low-field NMR setup to exploit the relatively short dead time of the receiver compared to high-resolution solution-state NMR spectrometers. Nevertheless, a magic sandwich echo (MSE) sequence was used to overcome the dead time of around 15 µs obtaining a refocused FID in the time frame of 1.6 µs to 200 µs with only minor intensity losses. This enables monitoring the fast relaxation of rigid components. In the time range of 15 µs to 200 µs a free induction decay (FID) can be used for observing transverse relaxation, whereas for the detection of the slow relaxing components, an additional time-incremented Hahn-Echo (HE) was measured to avoid long-term distortions due to B_0 field inhomogeneities. Upon fitting, the FID needs to be weighted by a factor of 10% to account for the significantly higher number of data points. The subsequent data processing procedure is listed below:

Intensity of HE scaled to match the FID intensities (FID+HE, red dots)

Tail Fit (dark gray) of HE with the following fit function (equation S2) to describe the T_2 relaxation time and share of the very mobile aqueous component

$$y = A_w exp\left\{-\left(\frac{t}{T_{2w}}\right)\right\} \quad \text{(S3)}$$

Subtracting the T_2 relaxation of the aqueous component from the FID+HE decays to reduce it to the polymeric moieties (reduced FID+HE, blue triangles)

Global Fit od MSE and FID using a two-component fit function (equation S3) to reliably describe the most rigid component

$$y = A_1 exp\left\{-\left(\frac{t}{T_{2rig}}\right)^2\right\} + A_2 exp\left\{-\left(\frac{t}{T_2}\right)\right\} \quad \text{(S4)}$$

$$T_{2rig} < T_2$$

Transferring the T_2 relaxation time of the most rigid component (T_{2rig}) to a three-component global fit of MSE and FID+HE to obtain the intermediate (T_{2int}) and mobile moieties (T_{2mob})

$$y = A_3 exp\left\{-\left(\frac{t}{T_{2rig}}\right)^2\right\} + A_4 exp\left\{-\left(\frac{t}{T_{2int}}\right)\right\} + A_5 exp\left\{-\left(\frac{t}{T_{2mob}}\right)\right\} \quad \text{(S5)}$$

$$T_{2rig} < T_{2int} < T_{2mob}$$

$$\frac{A_3}{A_3+A_4+A_5} = A_{rig} ; \frac{A_4}{A_3+A_4+A_5} = A_{int} ; \frac{A_5}{A_3+A_4+A_5} = A_{mob}$$

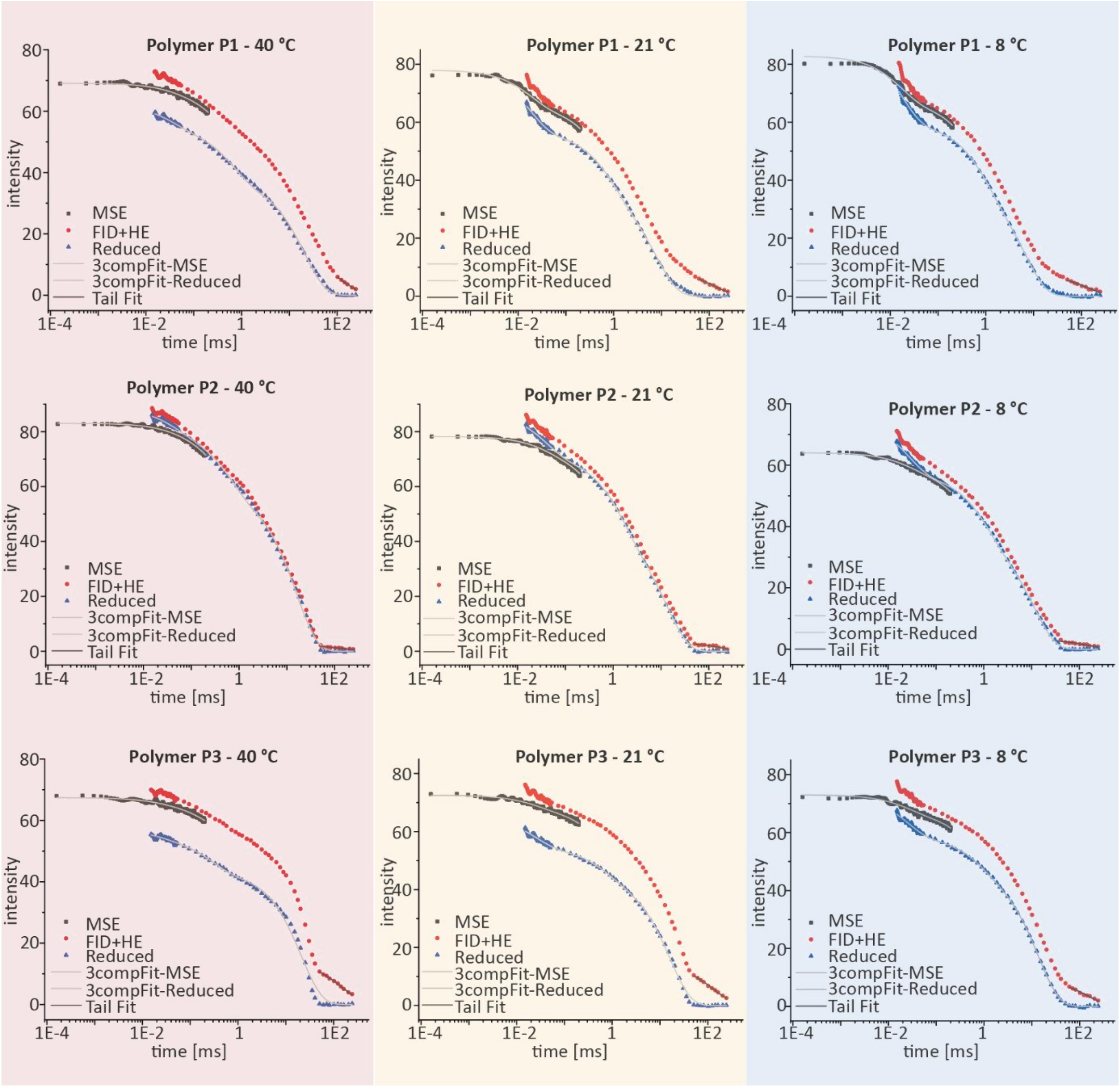

Figure S6.1 Free Induction Decay (FID), Magic Sandwich Echo (MSE) and Hahn-Echo (HE) decays of 20 wt.% samples of **P1**, **P2** and **P3** in D_2O at 8°C, 21°C and 40°C measured at a Bruker minispec mq20 at 20 MHz. The Tail Fit of combined FID and Hahn-Echo (FID+HE, red dots) referring to the very mobile water content of the sample is plotted in dark gray. Subtracting the aqueous component of FID+HE results in a reduced FID+HE version (blue triangles). Global fits of the Magic Sandwich Echo (MSE, gray squares) and reduced FID+HE decays are illustrated in light gray.

Double quantum (DQ) NMR experiments were measured of 20 wt.% samples of **P1**, **P2** and **P3** in D_2O at 8°C, 21°C and 40°C and evaluated following published procedures.[36-37] As shown on the left side of Figures S5-S7 a DQ intensity build-up I_{DQ} as well as a reference intensity I_{ref} can be obtained dependent on the evolution time t_{DQ}. A DQ build-up directly reflects the presence and the magnitude of residual dipolar couplings within the polymers, which in turn relates to constraints of the polymer strands. It is thus more qualitative than a simple T_2 measurement. The reference intensity contains information about the mobile fractions within the sample and can be used to correct the DQ build-up for relaxation effects. A detailed description of the data processing procedure is subsequently given:

Tail fit of I_{ref} -I_{DQ} with a two-component fit function (equation S3) starting at excitation time t_{DQ} = 2 ms and at t_{DQ} = 6 ms

Subtracting the two mobile components to yield $I_{\Sigma MQ}$ according to the following equation S5:

$$I_{\Sigma \mathrm{MQ}} = I_{ref} + I_{DQ} - A_1 \exp\left(\frac{-\tau_{DQ}}{T_2^1}\right) + A_2 \exp\left(\frac{-\tau_{DQ}}{T_2^2}\right) \tag{S6}$$

Normalization of the DQ build-up:

$$I_{\mathrm{nDQ}} = \frac{I_{DQ}}{I_{\Sigma MQ}} \tag{S7}$$

Fitting of I_{nDQ} to obtain lognormally distributed residual dipolar coupling constants (D_{res})

Two mobile components are obtained by tail fitting I_{ref} -I_{DQ} with a two-component fit function but depending on the starting point, different fit values were obtained indicating a smooth transition from more rigid to more mobile polymer strands. To reflect this gradual behavior fit values of both fits were subsequently used for further data evaluation yielding lognormally distributed residual dipolar coupling constants (D_{res}).

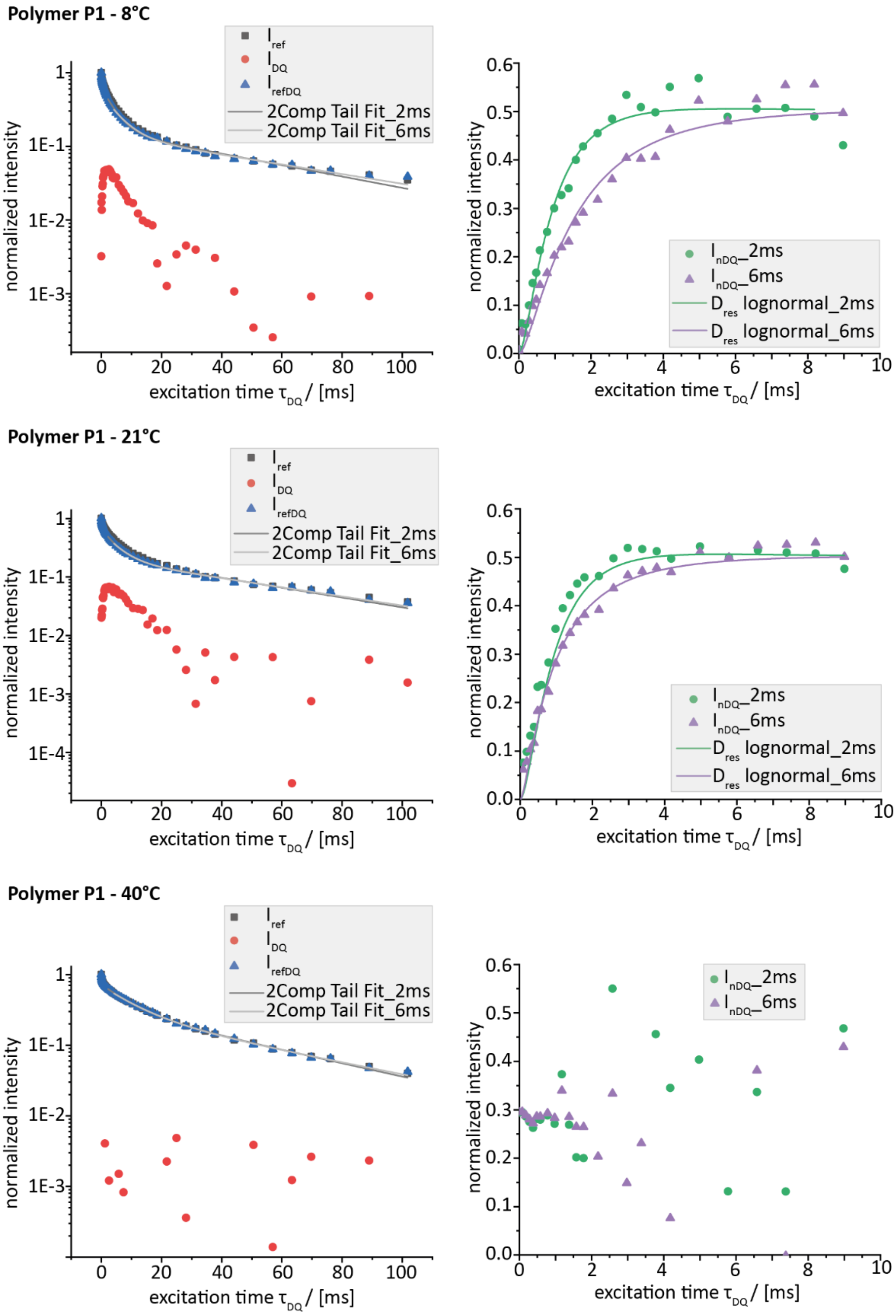

Figure S6.2 Double quantum (DQ) NMR build-up and decay measured for a 20 wt.% sample of **P1** in D2O at 8°C, 21°C and 40°C. On the left, the as-acquired reference intensity (I_{ref}, black squares) and double quantum intensity (I_{DQ}, red dots) and $I_{refDQ} = I_{ref} - I_{DQ}$ (blue triangles) are plotted. Biexponential tail fits of I_{refDQ} starting at an excitation time t_{DQ} = 2 ms and t_{DQ} = 6 ms are plotted in light gray and dark gray respectively. On the right side DQ build-ups are shown after subtracting the mobile fractions obtained of fitting after 2 ms (I_{nDQ}_2ms, green dots) or 6 ms (I_{nDQ}_6ms, purple triangles) with the respective fit yielding lognormally distributed residual dipolar coupling constants (D_{res}).

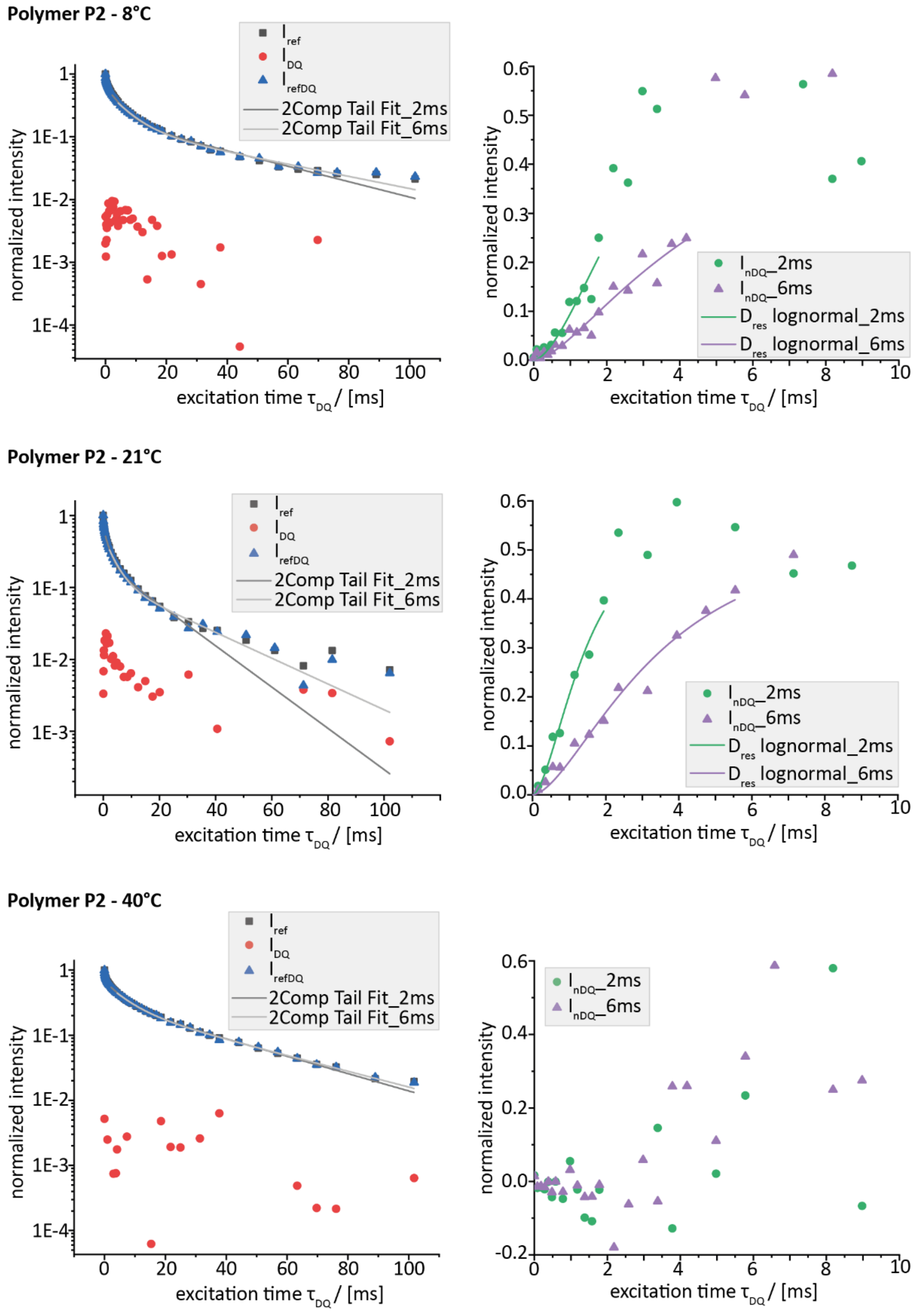

Figure S6.3 Double quantum (DQ) NMR build-up and decay measured for a 20 wt.% sample of **P2** in D_2O at 8°C, 21°C and 40°C. On the left, the as-acquired reference intensity (I_{ref}, black squares) and double quantum intensity (I_{DQ}, red dots) and $I_{refDQ} = I_{ref} - I_{DQ}$ (blue triangles) are plotted. Biexponential tail fits of I_{refDQ} starting at an excitation time t_{DQ} = 2 ms and t_{DQ} = 6 ms are plotted in light gray and dark gray respectively. On the right side DQ build-ups are shown after subtracting the mobile fractions obtained of fitting after 2 ms (I_{nDQ}_2ms, green dots) or 6 ms (I_{nDQ}_6ms, purple triangles) with the respective fit yielding lognormally distributed residual dipolar coupling constants (D_{res}).

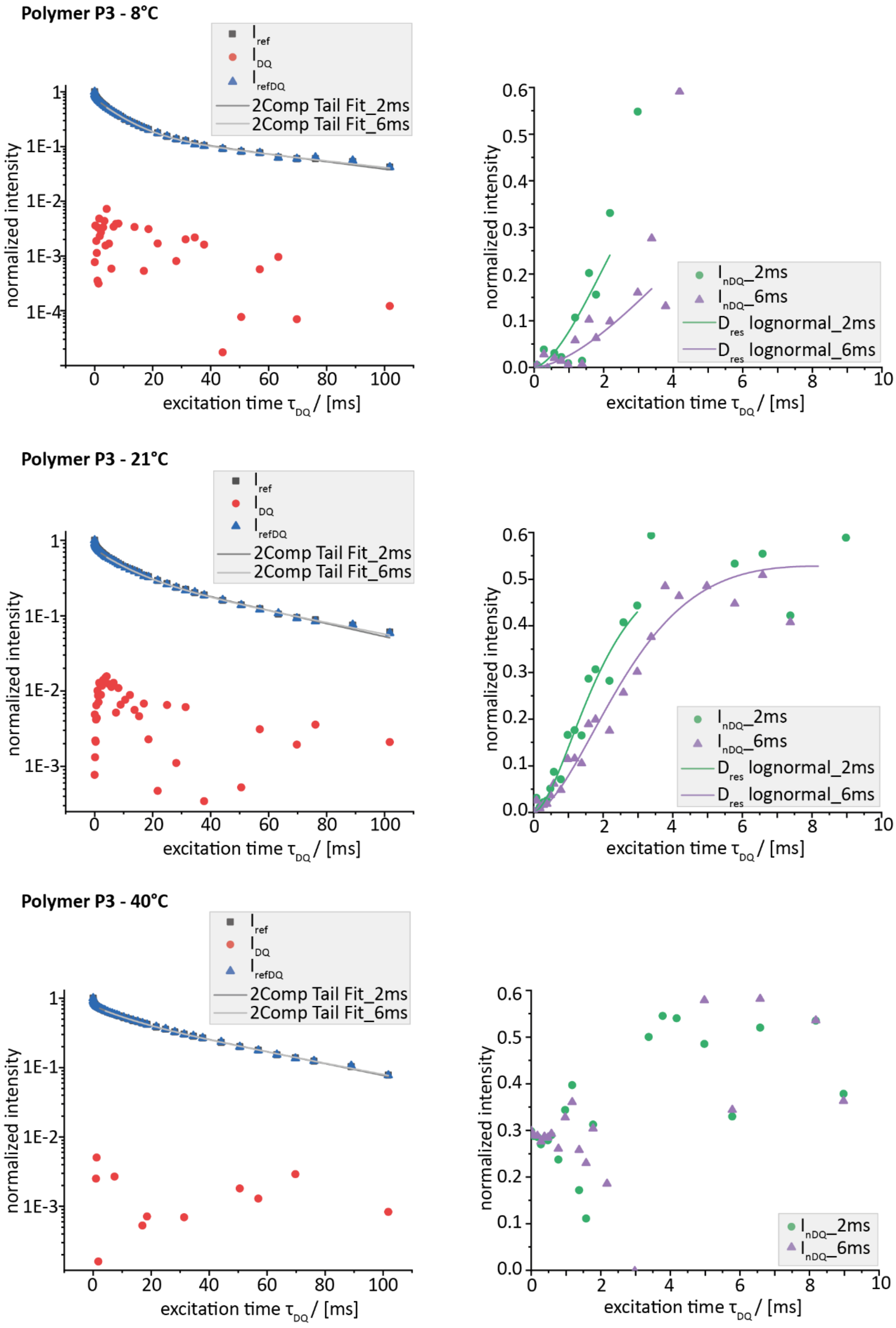

Figure S6.4 Double quantum (DQ) NMR build-up and decay measured for a 20 wt.% sample of **P3** in D_2O at 8°C, 21°C and 40°C. On the left, the as-acquired reference intensity (I_{ref}, black squares) and double quantum intensity (I_{DQ}, red dots) and $I_{refDQ} = I_{ref} - I_{DQ}$ (blue triangles) are plotted. Biexponential tail fits of I_{refDQ} starting at an excitation time t_{DQ} = 2 ms and t_{DQ} = 6 ms are plotted in light gray and dark gray respectively. On the right side DQ build-ups are shown after subtracting the mobile fractions obtained of fitting after 2 ms (I_{nDQ}_2ms, green dots) or 6 ms (I_{nDQ}_6ms, purple triangles) with the respective fit yielding lognormally distributed residual dipolar coupling constants (D_{res}).

Chapter S7 – Solid-state NMR experiments

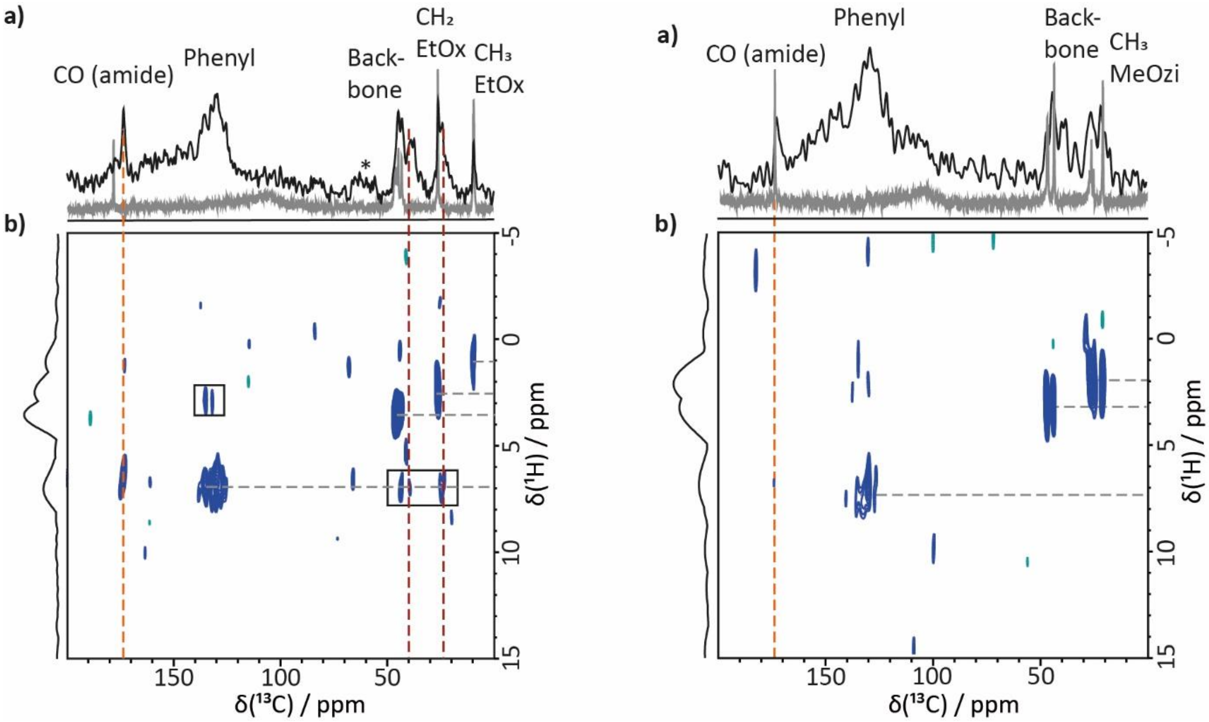

Figure S7 Left side: 20 wt.% sample of **P2** in D_2O; Right side: 20 wt.% sample of **P3** in D_2O a) Overlay of the ^{13}C NMR spectra recorded at 9.4 T and 7 kHz MAS using DE and short interscan delay of 1 s (gray) or CP MAS with 2 ms contact time (black). Spinning sidebands are indicated by asterisks. b) ^{1}H-^{13}C HETCOR MAS spectrum recorded at 9.4 T and a MAS rate of 7 kHz using a contact time of 2 ms. 140 (**P2**) or 82 (**P3**) t_1 FID increments were acquired using a recycle delay of 1.3 s, each with 800 (**P2**) or 1024 (**P3**) co-added transients. Direct CH contacts are indicated by dotted gray lines.

Chapter S8 – Molecular modeling

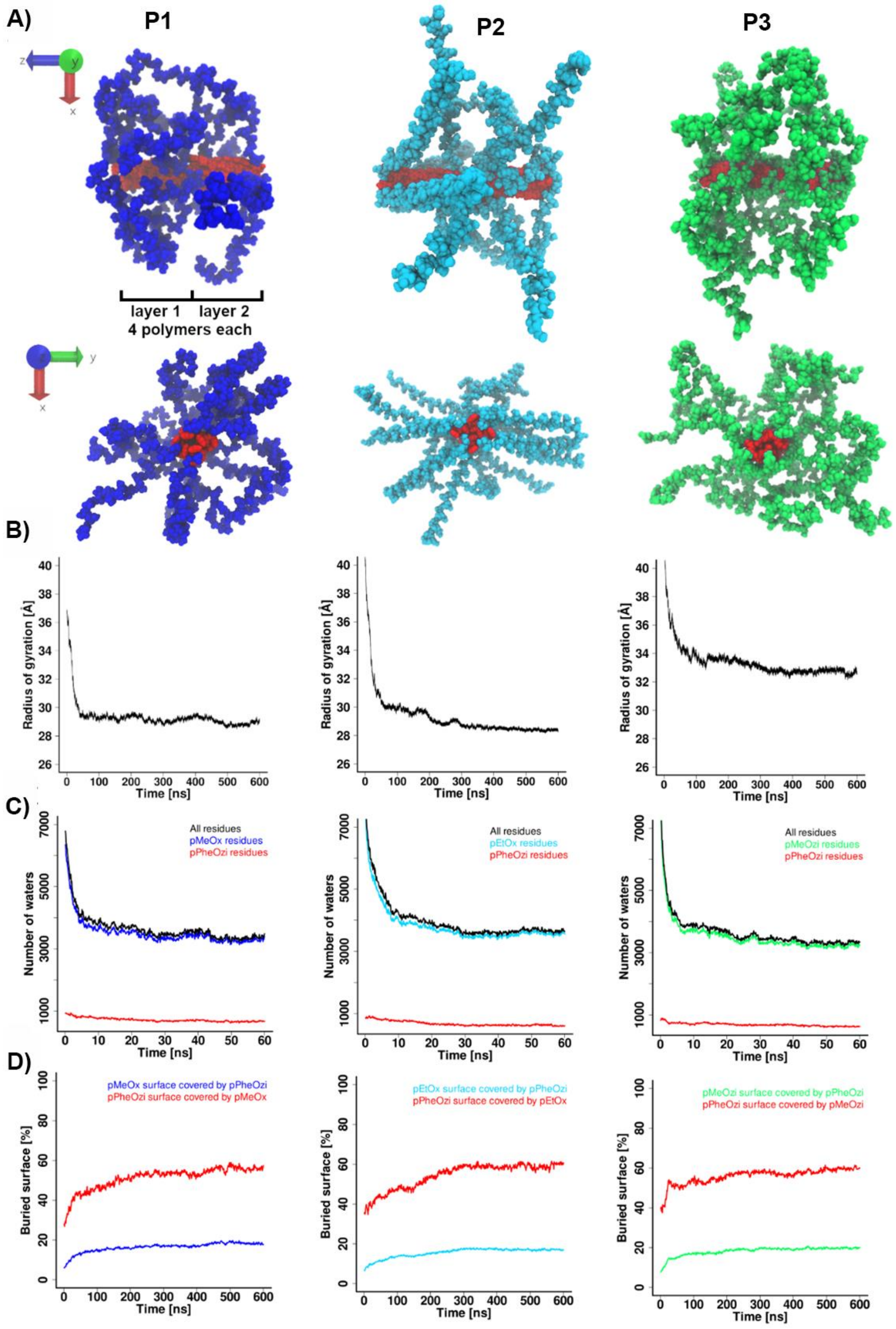

Figure S8.1 A) Illustration of starting structures for the MD simulations, shown from two angles. Hydrophobic pPheOzi blocks are depicted as red and hydrophilic A-blocks as blue van-der-Waals spheres. Axes are depicted in the lower left corner (z axis = blue arrow). B) Calculated radii of gyration of polymer heavy atoms. C) Number of water molecules within 5 Å of polymer residues. D) Fractions of surface areas covered by A and pPheOzi blocks respectively.

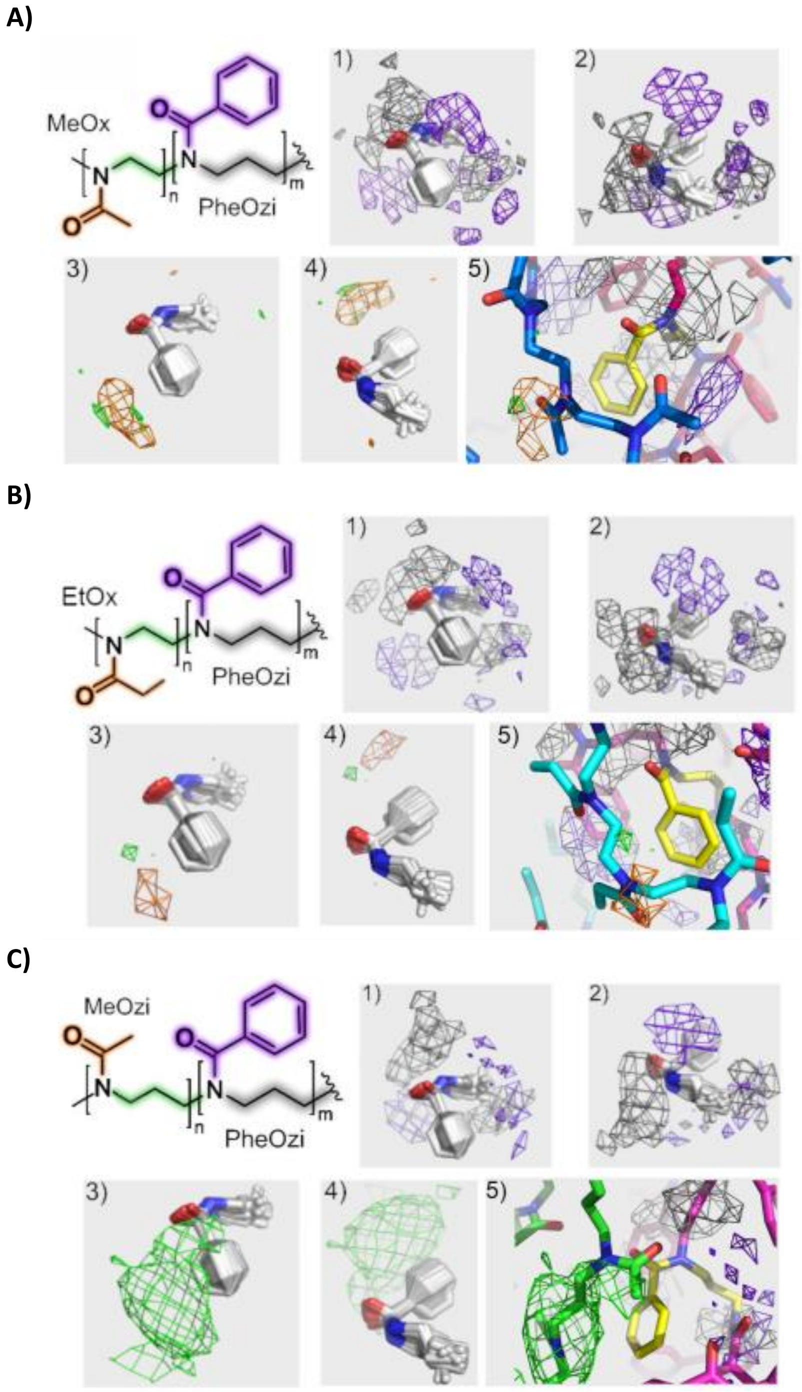

Figure S8.2 Results of molecular modeling of worm-like micelles comprising **P1** (A), **P2** (B) or **P3** (C). Occupancy density analyses around aligned pPheOzi residues (white sticks), showing hotspots for different polymer structures as meshes from two different perspectives. In 1) and 2) the violet densities represent pPheOzi side chains and the gray densities pPheOzi backbone atoms (isovalues: 0.08). Structures in 3) and 4) depict densities (isovalues: 0.03) for A-block backbone atoms (green) and side chain atoms (orange) from two different perspectives. 5) illustrates an exemplary MD snapshot in which residues at the surface of the micelle overlap with occupancy hotspots. Herein, A-blocks are shown with blue, turquoise and green carbon atoms, respectively, pPheOzi residues with magenta carbon atoms, and the aligned monomer of interest is highlighted in yellow. Densities are shown analogously as in the illustrations 1) to 4).

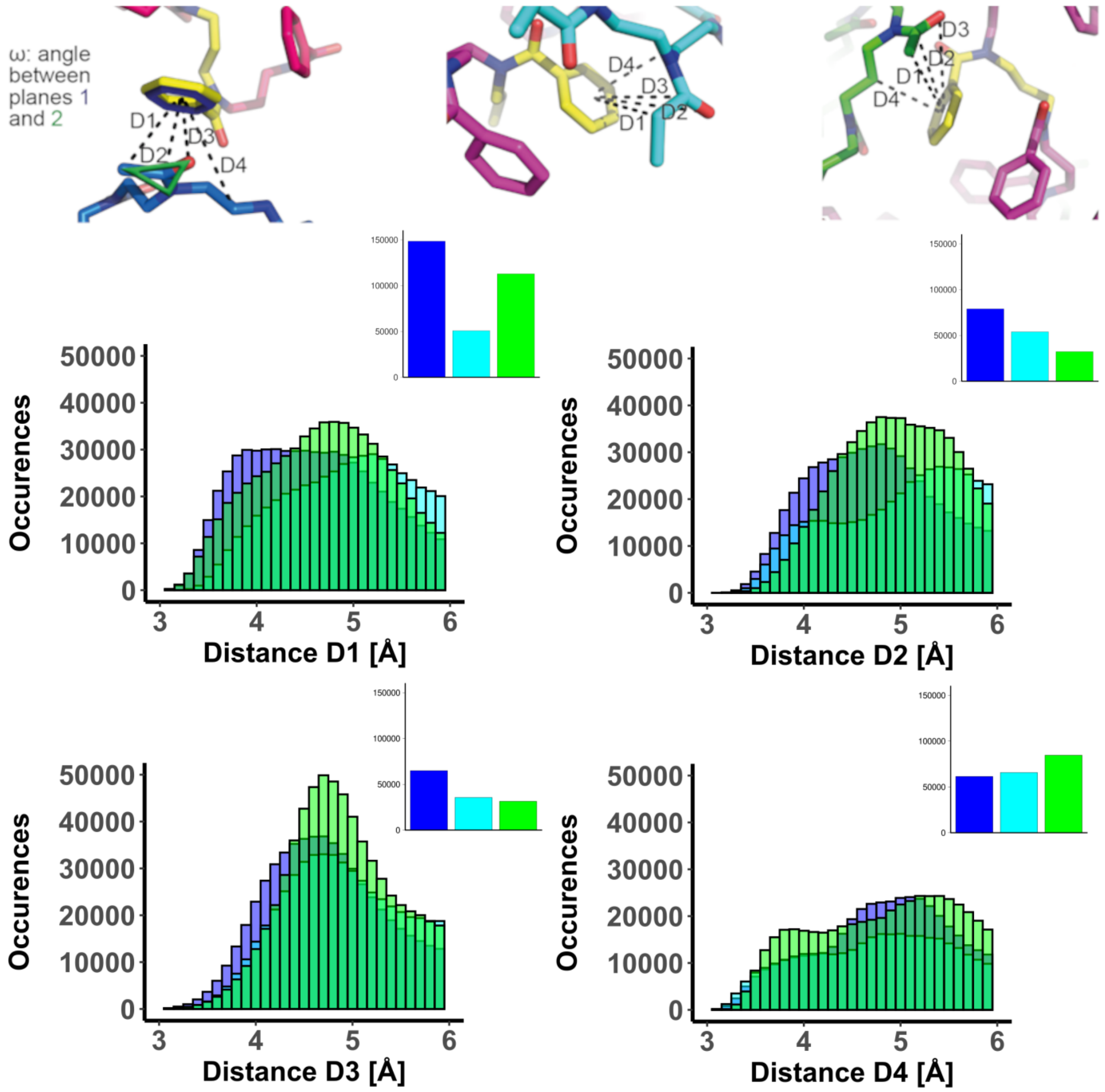

Figure S8.3 Histograms (bin size: 0.1 Å) for all distances up to 6 Å between pPheOzi moieties and the nearest polymer atoms (excluding hydrogen atoms). Dark blue describes P1, light blue P2 and green P3. Plots show the total amount of occurrences for all snapshots of all 104 pPheOzi residues which were used for density calculation. Barplots next to each histogram further compare the total amount of occurrences below 4 Å for each polymer type.

Present Addresses:

#J.K.: Soft Matter Chemistry, Department of Chemistry, Faculty of Science, Helsinki University, A. I. Virtasen Aukio 1, 00014 Helsinki, Finland

$B.S.: Deutsches Elektronen-Synchrotron DESY, Notkestr. 85, 22607 Hamburg, Germany

References

[1] Witte, H.; Seeliger, W. Cyclische Imidsäureester Aus Nitrilen Und Aminoalkoholen *Liebigs Ann. Chem.* **1974**, *1974*, 996-1009.

[2] Dreiss, C. A.; Jack, K. S.; Parker, A. P. On the Absolute Calibration of Bench-Top Small-Angle X-Ray Scattering Instruments: A Comparison of Different Standard Methods *J. Appl. Crystallogr.* **2006**, *39*, 32-38.

[3] Zhang, F.; Ilavsky, J.; Long, G. G.; Quintana, J. P. G.; Allen, A. J.; Jemian, P. R. Glassy Carbon as an Absolute Intensity Calibration Standard for Small-Angle Scattering *Metall. Mater. Trans. A* **2010**, *41*, 1151-1158.

[4] Pauw, B. R.; Smith, A. J.; Snow, T.; Terrill, N. J.; Thunemann, A. F. The Modular Small-Angle X-Ray Scattering Data Correction Sequence *J. Appl. Crystallogr.* **2017**, *50*, 1800-1811.

[5] Levitt, J. A.; Kuimova, M. K.; Yahioglu, G.; Chung, P.-H.; Suhling, K.; Phillips, D. Membrane-Bound Molecular Rotors Measure Viscosity in Live Cells Via Fluorescence Lifetime Imaging *J. Phys. Chem. C* **2009**, *113*, 11634-11642.

[6] Lisitsyna, E.; Efimov, A.; Depresle, C.; Cauchois, P.; Vuorimaa-Laukkanen, E.; Laaksonen, T.; Durandin, N. Deciphering Multiple Critical Parameters of Polymeric Self-Assembly by Fluorescence Spectroscopy of a Single Molecular Rotor Bodipy-C12 *Macromolecules* **2021**, *54*, 655-664.

[7] Dent, M. R.; López-Duarte, I.; Dickson, C. J.; Geoghegan, N. D.; Cooper, J. M.; Gould, I. R.; Krams, R.; Bull, J. A.; Brooks, N. J.; Kuimova, M. K. Imaging Phase Separation in Model Lipid Membranes through the Use of Bodipy Based Molecular Rotors *Phys. Chem. Chem. Phys.* **2015**, *17*, 18393-18402.

[8] Choe, C.; Lademann, J.; Darvin, M. E. Depth Profiles of Hydrogen Bound Water Molecule Types and Their Relation to Lipid and Protein Interaction in the Human Stratum Corneum in Vivo *Analyst* **2016**, *141*, 6329-6337.

[9] Unal, M.; Akkus, O. Shortwave-Infrared Raman Spectroscopic Classification of Water Fractions in Articular Cartilage Ex Vivo *J. Biomed. Opt.* **2018**, *23*, 1-11.

[10] Baum, J.; Pines, A. Nmr Studies of Clustering in Solids *J. Am. Chem. Soc.* **1986**, *108*, 7447-7454.

[11] Bond, S. D.; Leimkuhler, B. J.; Laird, B. B. The Nosé–Poincaré Method for Constant Temperature Molecular Dynamics *J. Comput. Phys.* **1999**, *151*, 114-134.

[12] Sturgeon, J. B.; Laird, B. B. Symplectic Algorithm for Constant-Pressure Molecular Dynamics Using a Nosé–Poincaré Thermostat *J. Chem. Phys* **2000**, *112*, 3474-3482.

[13] Maier, J. A.; Martinez, C.; Kasavajhala, K.; Wickstrom, L.; Hauser, K. E.; Simmerling, C. Ff14sb: Improving the Accuracy of Protein Side Chain and Backbone Parameters from Ff99sb *J. Chem. Theory Comput.* **2015**, *11*, 3696-3713.

[14] Gerber, P. R.; Müller, K. Mab, a Generally Applicable Molecular Force Field for Structure Modelling in Medicinal Chemistry *J. Comput. Aided Mol. Des.* **1995**, *9*, 251-268.

[15] Labute, P. The Generalized Born/Volume Integral Implicit Solvent Model: Estimation of the Free Energy of Hydration Using London Dispersion Instead of Atomic Surface Area *J. Comput. Chem.* **2008**, *29*, 1693-1698.

[16] Chemical Computing Group ULC. *MOE. Molecular Operating Environment (MOE),* 2019.01; 1010 Sherbook St. West, Suite #910, Montreal, QC, Canada, H3A 2R7, **2019**.

[17] Yan, H.; Han, Z.; Li, K.; Li, G.; Wei, X. Molecular Dynamics Simulation of the Ph-Induced Structural Transitions in Ctab/Nasal Solution *Langmuir* **2018**, *34*, 351-358.
[18] Wiest, J.; Kehrein, J.; Saedtler, M.; Schilling, K.; Cataldi, E.; Sotriffer, C. A.; Holzgrabe, U.; Rasmussen, T.; Böttcher, B.; Cronin-Golomb, M.; Lehmann, M.; Jung, N.; Windbergs, M.; Meinel, L. Controlling Supramolecular Structures of Drugs by Light *Mol. Pharmaceutics* **2020**, *17*, 4704-4708.
[19] Wang, Z.; Larson, R. G. Molecular Dynamics Simulations of Threadlike Cetyltrimethylammonium Chloride Micelles: Effects of Sodium Chloride and Sodium Salicylate Salts *J. Phys. Chem. B* **2009**, *113*, 13697-13710.
[20] Woods, R. J.; Chappelle, R. Restrained Electrostatic Potential Atomic Partial Charges for Condensed-Phase Simulations of Carbohydrates *J. Mol. Struct.: THEOCHEM* **2000**, *527*, 149-156.
[21] F Frisch, M. J.; Trucks, G. W.; Schlegel, H. B.; Scuseria, G. E.; Robb, M. A.; Cheeseman, J. R.; Scalmani, G.; Barone, V.; Petersson, G. A.; Nakatsuji, H.; Li, X.; Caricato, M.; Marenich, A. V.; Bloino, J.; Janesko, B. G.; Gomperts, R.; Mennucci, B.; Hratchian, H. P.; Ortiz, J. V.; Izmaylov, A. F.*, et al. Gaussian 09,* revision D.01; Gaussian, Inc.: Wallingford, CT, **2009**.
[22] Wang, J.; Wolf, R. M.; Caldwell, J. W.; Kollman, P. A.; Case, D. A. Development and Testing of a General Amber Force Field *J. Comput. Chem.* **2004**, *25*, 1157-1174.
[23] Case, D. A.; Cheatham III, T. E.; Darden, T.; Gohlke, H.; Luo, R.; Merz Jr., K. M.; Onufriev, A.; Simmerling, C.; Wang, B.; Woods, R. J. The Amber Biomolecular Simulation Programs *J. Comput. Chem.* **2005**, *26*, 1668-1688.
[24] Case, D. A.; Ben-Shalom, I. Y.; Brozell, S. R.; Cerutti, D. S.; Cheatham, T. E.; Cruzeiro, V. W. D.; Darden, T. A.; Duke, R. E.; Ghoreishi, D.; Gilson, M. K.; Gohlke, H.; Goetz, A. W.; Greene, D.; Homeyer, N.; Izadi, S.; Kovalenko, A.; Kurtzman, T.; Lee, T. S.; LeGrand, S.; Li, P.*, et al. AMBER 2018*; University of California: San Francisco, CA, **2018**.
[25] Jorgensen, W. L.; Chandrasekhar, J.; Madura, J. D.; Impey, R. W.; Klein, M. L. Comparison of Simple Potential Functions for Simulating Liquid Water *J. Chem. Phys* **1983**, *79*, 926-935.
[26] Phillips, J. C.; Braun, R.; Wang, W.; Gumbart, J.; Tajkhorshid, E.; Villa, E.; Chipot, C.; Skeel, R. D.; Kalé, L.; Schulten, K. Scalable Molecular Dynamics with Namd *J. Comput. Chem.* **2005**, *26*, 1781-1802.
[27] Darden, T.; York, D.; Pedersen, L. Particle Mesh Ewald: An N·Log(N) Method for Ewald Sums in Large Systems *J. Chem. Phys* **1993**, *98*, 10089-10092.
[28] Roe, D. R.; Cheatham, T. E., III Ptraj and Cpptraj: Software for Processing and Analysis of Molecular Dynamics Trajectory Data *J. Chem. Theory Comput.* **2013**, *9*, 3084-3095.
[29] Renouprez, A., in *Catal. Charact.*, Springer, **1994**, pp. 445-465.
[30] Hyland, L. L.; Taraban, M. B.; Yu, Y. B. Using Small-Angle Scattering Techniques to Understand Mechanical Properties of Biopolymer-Based Biomaterials *Soft Matter* **2013**, *9*, 10218-10228.
[31] Baral, A.; Basak, S.; Basu, K.; Dehsorkhi, A.; Hamley, I. W.; Banerjee, A. Time-Dependent Gel to Gel Transformation of a Peptide Based Supramolecular Gelator *Soft Matter* **2015**, *11*, 4944-4951.
[32] Kruszynski, R.; Sierański, T. Can Stacking Interactions Exist Beyond the Commonly Accepted Limits? *Cryst. Growth Des.* **2016**, *16*, 587-595.
[33] Manna, A.; Chakravorti, S. Switching of Emission of a Styryl Dye in Cucurbit[7]Uril: A Comprehensive Experimental and Theoretical Study *Spectrochim. Acta, Part A* **2015**, *140*, 241-247.
[34] Vyšniauskas, A.; López-Duarte, I.; Duchemin, N.; Vu, T.-T.; Wu, Y.; Budynina, E. M.; Volkova, Y. A.; Peña Cabrera, E.; Ramírez-Ornelas, D. E.; Kuimova, M. K. Exploring Viscosity, Polarity and Temperature Sensitivity of Bodipy-Based Molecular Rotors *Phys. Chem. Chem. Phys.* **2017**, *19*, 25252-25259.
[35] Boireau-Adamezyk, E.; Baillet-Guffroy, A.; Stamatas, G. N. Mobility of Water Molecules in the Stratum Corneum: Effects of Age and Chronic Exposure to the Environment *J. Invest. Dermatol.* **2014**, *134*, 2046-2049.
[36] Höpfner, J.; Guthausen, G.; Saalwächter, K.; Wilhelm, M. Network Structure and Inhomogeneities of Model and Commercial Polyelectrolyte Hydrogels as Investigated by Low-Field Proton Nmr Techniques *Macromolecules* **2014**, *47*, 4251-4265.

[37] Saalwächter, K.; Gottlieb, M.; Liu; Oppermann, W. Gelation as Studied by Proton Multiple-Quantum Nmr *Macromolecules* **2007**, *40*, 1555-1561.

Author Contributions

Conceptualization: L.H., R.L.

Funding acquisition and resources: R.L., A.-C.P.

Formal analysis and investigation: L.H., T.Z., J.K., T.K., A.-L. Z., S.F., B.S., E.L., N.D.

Methodology: K.S.

Project administration: L.H., T.Z., R.L., A.-C.P.

Supervision: T.L., C.S., K.S., M.W., R.L., A.-C.P.

Visualization: L.H., T.Z.

Writing – original draft: L.H., T.Z.

Writing -review & editing: L.H., T.Z., R.L., A.-C.P.

All authors have read and agreed to the published version of the manuscript.
